# Modeled changes of cerebellar activity in mutant mice are predictive of their learning impairments

**DOI:** 10.1038/srep36131

**Published:** 2016-11-02

**Authors:** Aleksandra Badura, Claudia Clopath, Martijn Schonewille, Chris I. De Zeeuw

**Affiliations:** 1 Netherlands Institute for Neuroscience, Royal Dutch Academy of Arts & Sciences, Amsterdam, The Netherlands; 2 Princeton Neuroscience Institute and Department of Molecular Biology, Princeton University, Princeton, NJ, USA; 3 Bioengineering Department, Imperial College London, UK; 4 Department of Neuroscience, Erasmus MC, Rotterdam, The Netherlands

## Abstract

Translating neuronal activity to measurable behavioral changes has been a long-standing goal of systems neuroscience. Recently, we have developed a model of phase-reversal learning of the vestibulo-ocular reflex, a well-established, cerebellar-dependent task. The model, comprising both the cerebellar cortex and vestibular nuclei, reproduces behavioral data and accounts for the changes in neural activity during learning in wild type mice. Here, we used our model to predict Purkinje cell spiking as well as behavior before and after learning of five different lines of mutant mice with distinct cell-specific alterations of the cerebellar cortical circuitry. We tested these predictions by obtaining electrophysiological data depicting changes in neuronal spiking. We show that our data is largely consistent with the model predictions for simple spike modulation of Purkinje cells and concomitant behavioral learning in four of the mutants. In addition, our model accurately predicts a shift in simple spike activity in a mutant mouse with a brainstem specific mutation. This combination of electrophysiological and computational techniques opens a possibility of predicting behavioral impairments from neural activity.

The cerebellum is crucial for motor and sensory integration. One of the best-studied functions of the cerebellum is motor learning and yet despite a large body of behavioral and electrophysiological data on cerebellar motor adaptation, underlying processes of sensory integration remain elusive. Based on recent advances in experimental methods it has been shown that cerebellar learning encompasses multiple sites of plasticity in cerebellar cortex as well as in the deep nuclei^1^,^2^. This new insight into cerebellar physiology allowed expansion of classical cerebellar learning models, which stated that learning in the cerebellum involves exclusively plasticity at the granule cell (GC) to Purkinje cell (PC) synapse guided by the climbing fiber (CF) input, acting as the teaching signal^3^,^4^,^5^.

Recently, we have shown that a model encompassing bidirectional plasticity at the GC-PC synapse supervised by CFs^6^, PC inhibition by molecular layer interneurons (MLIs)^7^,^8^ as well as plasticity at the mossy fiber (MF) to medial vestibular nuclei (MVN) synapse^9^,^10^ (Fig. 1a) can reliably reproduce empirical data of the vestibulo-ocular reflex (VOR) phase-reversal task. During this task the phase of the compensatory eye movements with respect to sinusoidal stimulation of a turntable is reversed over the course of several days by providing an in-phase visual stimulation (Fig. 1b) 11. Traditionally, VOR adaptation was studied predominantly using paradigms in which only the gain of the eye movements was increased or decreased^12^,^13^,^14^,^15^. Although these paradigms are informative and have revealed a lot of information about the underlying circuitry controlling the VOR reflex, their application allows for studying only one phenomenon at the time. The VOR phase reversal paradigm, for which we created our model, consists of the ‘classic’ VOR gain decrease on the first day, followed by the phase reversal training that requires changes in phase, but also features a correlated later increase in gain. Therefore it effectively binds multiple elements in a single paradigm that is applied throughout multiple days of training, during which the phase and gain changes are consolidated^16^. It is therefore very robust and sensitive to small disruptions in cerebellar circuitry. Both gain and phase adaptations of the VOR require the cortex of the vestibulocerebellum and both use the same pathways to convey the visual, vestibular and oculomotor input signals as well as the same oculomotor pathways to control the oculomotor output^15^,^17^,^18^. Furthermore, effects of genetic aberrations of cerebellar cortical neurons can most prominently be revealed by multiple day training paradigms^13^,^16^,^19^,^20^. Indeed, compensatory mechanisms have mostly been found insufficient to occlude the outcome of a genetic lesion in the case of VOR phase-reversal learning^16^,^19^,^20^,^21^,^22^. We speculate that this is due to the fact that this form of motor learning requires all sites of plasticity to be intact and to work in synergy to produce the desired change in motor output^2^. Importantly, unlike more complex motor behaviors such as gait adaptation or eye-blink conditioning, VOR adaptation involves neither higher cortical areas nor thalamic nuclei, largely restricting degrees of freedom whilst modeling.

Previously, using electrophysiological and behavioral data from wild type mice and behavioral data from two cerebellar cell-specific mutant mice that both show impairments in the VOR phase-reversal task^16^,^20^, we were able to build and verify a novel mechanistic model, accounting for the observed changes^11^. These mutant mice included the *PC*-*Δγ2* mice, in which the γ2 subunit of the GABAA receptor is specifically removed from the PCs using the L7-Cre-lox system^16^, and the *GC*-*ΔKCC2* mice, in which the co-transporter KCC2 is knocked out of GCs using the A6-Cre-lox system^20^. In addition, our model allowed for a number of experimentally testable predictions about the neural coding underlying the mechanism of VOR phase-reversal adaptation. Specifically, it predicts that impaired VOR adaptation in mice lacking inhibition from MLIs to PC, such as the *PC*-*Δγ2* mice, or mice suffering from increased excitability of their GCs, such as the *GC*-*ΔKCC2* mice, should be directly reflected in changes in PC spiking rate and temporal patterns, otherwise referred to as PC modulation.

PCs elicit two very distinctive types of action potentials – complex-spikes (CSs) and simple-spikes (SSs)^3^,^23^. CSs are triggered exclusively by activation of CFs that originate in the inferior olive (IO)^24^. Baseline SS activity is intrinsic and in the absence of physical stimulation, PCs fire steadily at approximately 50–90 Hz dependent on their molecular identity (zebrin-positive and negative PCs)^25^. However, the SS patterns can be prominently influenced by excitation arising from parallel fibers (PFs)^19^,^26^ and inhibition from MLIs^27^. Sensorimotor information coming from CFs together with signals from PFs modulate the firing frequency and temporal patterns of both CSs and SSs, often resulting in reciprocal firing^22^. This means that when CS firing frequency increases, SS activity is attenuated, and *vice versa*. This phenomenon of reciprocity is particularly evident in the flocculo-nodular lobe during natural periodic, visual and vestibular stimulation, when SS activity oscillates between on–phase and off–phase firing frequencies of ~150 Hz and ~10 Hz, respectively^28^,^29^,^30^. During VOR stimulation in wild type mice SSs are modulated in anti-phase with ipsiversive head movement and this anti-phase modulation persists after VOR phase-reversal training, albeit at a different amplitude^11^.

Here we test the predictions of our model by performing *in vivo* electrophysiological recordings from PCs before and after VOR phase-reversal training in the *PC*-*Δγ2* and *GC*-*ΔKCC2* mutant mice^16^,^20^ and quantifying the overlap between the model and experimental data. Moreover, in order to have a complete picture of the cerebellar circuit and test the limits of our model we investigate the PCs spiking behavior in two additional mutant mice with Purkinje cell specific lesions. These include the *PC*-*ΔKCC2* mice^20^, in which the GABAergic inhibition of MLIs on PCs is significantly reduced, and the *PC-ΔPP2B* mice, in which long-term potentiation (LTP) at the PF-PC synapse is abolished and intrinsic excitability of PCs is reduced^21^. Both of these latter two mouse lines also use the Cre-lox system with the PC-specific L7-promoter ensuring that the deletion is limited to PCs only. In addition, we tested to what extent our model reproduces motor learning deficits resulting from major reduction of input from the granule cell layer, using a granule cell specific mutant *GC-ΔCACNA1A*, in which the majority of granule cells is silenced^19^. This mouse model utilizes an imperfect Cre-lox system together with a cerebellar GC-specific promoter^31^,^32^, resulting in a loss of GC output in an estimated ~75% of the GC population^19^. Notably, this decreased output from the GC layer results in loss of bidirectional plasticity at the PF to PC synapse^19^. Finally, we tested whether our model is consistent with the profound performance deficits and dramatic changes in PC activity in *IO-ΔRobo3* mice, in which the majority of CFs is unable to cross the midline and therefore project from the ipsilateral part of the IO^22^,^33^. This mouse line utilizes the PTF1a promoter to delete the *Robo3* gene specifically from the IO during a restricted time window in early development. Since Robo3 is a critical axonal targeting protein required for midline crossings^33^, its deletion largely prevents CFs to innervate PCs on the contralateral side. We show that our model is to a large extent able to reproduce the experimental results and accurately predicts the behavioral deficits in four out of six mutants, namely in the *GC*-*ΔKCC2, PC*-*Δγ2*, *PC*-*ΔKCC2*, and *IO-ΔRobo3* mice. The model failed to capture the behavioral and, to a large extent, electrophysiological data from *PC-ΔPP2B* mice and *GC*-*ΔCACNA1A* mice, which suggests a specific and critical role for additional compensatory sites of plasticity, such as at the GC to MLI synapse and/or for additional potential sites of deficits, such as at the PC to MVN synapse that were not included in the model. These additional sites of plasticity have not been tested in either of the two mutants. Together, these data highlight the role of SS modulation amplitude in cerebellar cortex dynamics during phase-shift paradigms.

## Results

The VOR is a form of compensatory eye movements, which produces eye movements in the opposite direction to the movement of the head, stabilizing the image on the retina. This basic reflex originates in the semicircular canals, where the hair cells detect head movements and send information on rotational acceleration to several vestibular nuclei (VN) in the brainstem. A major step of integration occurs in the prepositus hypoglossi (for the vertical axis) and the interstitial nucleus of Cajal (for both horizontal axes), where position and velocity signals of the head are processed^34^. This information is conveyed via the oculomotor nuclei onto the eye muscles and triggers the compensatory movement of the eyeball. However, due to the fact that at the level of brainstem VOR operates without any feedback, changes in its internal parameters (for example changes in the size of the eye ball or the strength of the orbital muscles due to aging) will cause errors in stabilization of the visual image. In order for this system to maintain its accuracy it needs a mechanism that will correct for possible errors and enable the VOR to remain properly calibrated. The cerebellum fulfills this role^18^,^35^. By combining the vestibular information with the visual input it provides the VOR with the error–correction system enabling adaptation. VOR adaptation is therefore a cerebellar dependent form of motor learning, which can be readily studied and manipulated in a laboratory setting.

**Figure 1:**
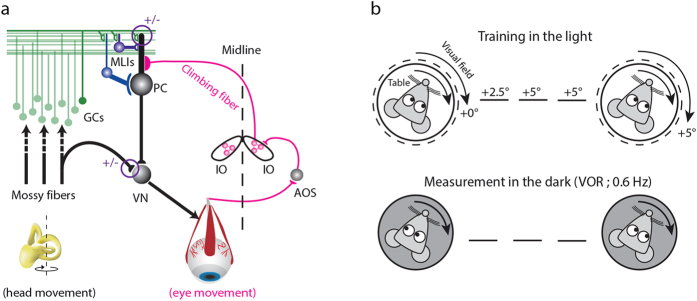
Cerebellar circuitry and experimental design. (**a**) Organization of a basic cerebellar module of the oculomotor pathway. Vestibular input to the vestibular nucleus (VN) carries the signal about the head movement (mossy fibers, black). This signal is also relayed by mossy fibers onto granule cells (GC, green), which innervate Purkinje cells (PC, black). The inferior olive (IO) receives information about the retinal slip that is first processed by the accessory optic system (AOS). The IO neurons innervate the contralateral PCs through climbing fibers (CF; purple). Those two inputs converge on PCs, which send their output back to VN, forming a loop. This loop is modulated by an inhibitory side loop represented by molecular layer interneurons (MLIs, blue). The magenta circles and +/− signs indicate sites of plasticity incorporated in the model. (**b**) Schematic representation of vestibulo-ocular response (VOR) phase-reversal adaptation. During the learning, a mouse is headfixed on a turntable and phase adaptation is achieved by an in-phase table and drum rotation in the light. With each of the five training sessions there is an increase in amplitude of the drum rotation, but the oscillation frequency of the turntable remains fixed at 0.6 Hz. Note that by the end of day five the phase of eye movements of a mouse is reversed so that the eye movements are now in-phase with the rotation of the turntable.

In our study, we quantified the firing behavior of PCs in the flocculus of several cell-specific mutant mice before and after application of phase-reversal training, a long–term adaptation paradigm aimed at shifting the phase of the eye movements during the VOR (Fig. 1b). Using our model we tested how the observed changes in PC activity in the mutants related to the spiking patterns in wild type mice and whether they were predictive of the impairments in behavior.

### Establishing the baseline – modeled and experimental phase-reversal training in control mice

**Figure 2:**
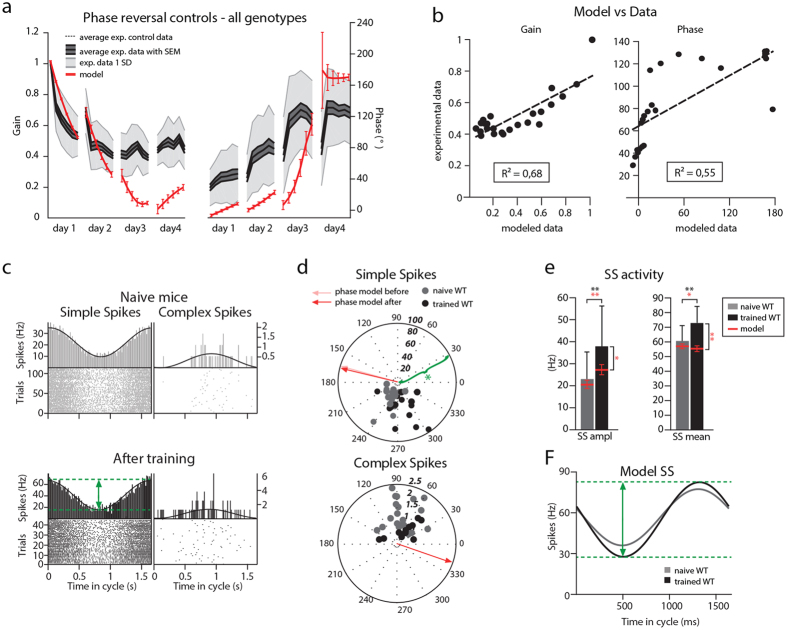
Experimental and modeled phase-reversal in control mice. (**a**) Experimental and modeled eye movements in control mice as a function of training time of VOR phase-reversal (training is done in the light and eye measurement is done in the dark during VOR at 0.6 Hz). Gain values (left panel) are normalized to the initial gain. Experimental data represent averages with SEM (dark area) and SD (light grey area) of all control mice used in this study. Modeled changes are displayed for both gain and phase with SD (red line). (**b**) Linear regression plots displaying correlation between modeled and experimental data. (**c**) Example cell of an *in vivo* extracellular recording from floccular vertical axis (VA) PCs obtained during vestibular stimulation (0.6 Hz) in the dark before and after the VOR-reversal training in wild type mice (top, grey and bottom, black panels, respectively) plotted as peri-stimulus time histograms (PSTH). Green arrows indicate the depth of the modulation in the trained animals (peak to peak, marked with a green dotted line). (**d**) Polar plots of SS (top) and CS (bottom) modulation before and after learning in control mice (grey and black, respectively). Phase of the modeled SS and CS are indicated with arrows. The amplitude of modulation is depicted by the radius (green asterisk; calculated by subtracting the trough of SS from the peak of the SS activity) and the phase of modulation is indicated by the angle. Each dot represents a single cell. (**e**) As predicted by the model (red) the SS modulation was significantly increased following learning in the control mice. There was also a small but significant increase in the SS firing frequency following the training. Error bars denote SD; *denote p < 0.05; **denote p < 0.001. (**f**) Modeled PC SS activity as a function of time in the cycle in wild type mice (grey, initial value before learning; black, after training). SS activity produced by the model was normalized to the mean average firing frequency of the PC in control mice. Green arrows indicate the depth of the modulation in the trained animals (peak to peak, marked with a green dotted line).

As previously described^11^, our model can predict changes in gain and phase values during the VOR phase-reversal training in wild type mice. Here we first measured and quantified to what extent the model could predict the behavioral changes of all littermate control mice used in the current study (Fig. 2a), (for details of all raw behavioral data, see: Wulff *et al.*^16^; Schonewille *et al.*^21^; Seja *et al.*^20^; Galliano *et al.*^19^)^16^,^19^,^20^,^21^,^22^. We re-analyzed all the raw data using circular statistics, and re-plotted the gain and phase values during the phase-reversal training. There were no significant differences between the speed and amount of gain changes during phase-reversal learning between all measured control groups (Supplementary Fig. 1, top panels; Supplementary Table 1). However, the maximal amount of phase shift in the littermate controls of the *GC*-Δ*CACANA* mice was significantly lower than that in the other littermate controls (Δ*KCC2* littermate controls, Δγ*2* littermate controls and Δ*PP2B* littermate controls; p = 0.006, one-way ANOVA; Supplementary Fig. 1, bottom panels; Supplementary Table 2). This possibly reflects the impact of slight differences in age^36^, breeding environment, or experimenters and experimental setups involved. Next, we quantified the correlation between the model and all averaged littermate controls. (Fig. 2b, n = 34 animals). The linear regression revealed strong correlation for the entire training for gain values (R^2^ = 0.68) and moderate correlation for the phase values (R^2^ = 0.55). The average distance between the modeled and experimental values, estimated using least-square distance, was 0.23 for gain and 57**°** for phase. On day-by-day basis the model predicted the gain very well for the first two days of training (day 1, R^2^ = 0.87; day 2, R^2^ = 0.72), but failed to capture the amplitude of the eye movements of days 3 and 4 (R^2^ = 0.05 and R^2^ = 0.08, respectively). Notably, despite this low correlation, the direction of the gain in experimental and modeled data were similar in that the gain dropped on day 3 and that it recovered on day 4. The correlation between the modeled and experimental phase values was also strong for days 1, 2 and 4 and low for day 3 (day 1, R^2^ = 0.86; day 2, R^2^ = 0.67; day 3, R^2^ = 0.29 and day 4, R^2^ = 0.98). Importantly, day 4 values showed a maximum shift in phase evoked by the VOR training and saturated at ~140**°** for experimental and ~160**°** for the modeled data.

Given the differences in the performance between littermate controls in the different mutant lines we also calculated the correlation between model and experimental data for each day, and total duration of the training, separately for each mutant line (Supplementary Table 3). Unsurprisingly, the strength of the correlation varied between the lines, but overall the same trend was observed as in the pooled control data where days 3 and 4 had the lowest correlation for gain values, whereas phase values were low for day 3 alone. Notably, the model consistently and accurately predicted the outcome of the training in that the first training session on day 4 had the largest standard deviation, that overall day 4 of the training showed a sharp increase in the phase shift, and that ultimately the phase saturated on day 4 reaching its maximum shift. Finally, consistent with the observations from our previous paper^11^, there was a shift in initial phase values between the model and experimental data of approximately 40**°** in all control lines.

Next, we analyzed spiking patterns obtained from *in vivo* recordings of PCs in Δ*KCC2* littermate controls and Δγ*2* littermate controls both before and after VOR training. We have also recorded cells during VOR in naïve Δ*PP2B* controls and Δ*CACANA1A* controls. We compared the frequencies, amplitudes and phases of SS and CS activity and found no significant differences between the control groups and data from the Black6 wild type mice discussed in our previous paper (Table 1). When measuring the cells after training we made sure that the phase of the eye movements was still reversed when compared to that of the naïve mice (Table 2). On average the mice maintained a phase of the eye movements of ~40° with respect to the turntable during the electrophysiological recordings performed before training and of ~140**°** after learning. The drop in the max phase shift was presumably a result of the delay between the last training session and first electrophysiological measurement, during which the mice were constantly kept in the dark (for details see Materials Methods). In all cells we have observed reciprocal modulation of SS and CS activity during VOR stimulation (Fig. 2c,d). The ~40**°** initial phase offset observed in the behavioral data translated to the offset in the phase of predicted PC spiking (Fig. 2d). As predicted, the average SS phase with respect to the head/table movements did not change following learning and averaged at 247 ± 7**°** in naïve mice, and 279 ± 6**°** in trained animals (p = 0.07). The model predicted phase of 163 ± 1**°** and 160 ± 1**°**, respectively (Table 1). However, when corrected for the initial offset of ~40**°** the modeled average of the phase values falls within 1 standard deviation of the experimental values. Notably, the model provided an accurate prediction of the SS amplitude of modulation for cells recorded in both the naïve animals and in the mice that underwent VOR training (Fig. 2e, Table 1). The amplitude increased significantly in both modeled and experimentally recorded data (P < 0.001 for both datasets). We speculate that the reason for the significant difference in the final modulation depth values between modeled and experimental SS activity can be attributed to large variability in SS spiking as well as simplistic modeling of the MLIs (see Discussion for details). Although the model correctly estimates the SS firing frequency in naïve mice, it does not capture the increase in SS activity following the training. The mean estimated value of the SS firing frequency was 56.9 ± 0.3 Hz for the naïve prediction and 55.3 ± 0.4 Hz in trained output (Fig. 2e). To allow for the readily comparison between mutants and control mice we normalized the values of the SS spiking produced by the model to the average SS frequency of the control cells (Fig. 2f).

### Impact of increased granule cell excitability on Purkinje cell modulation

**Figure 3:**
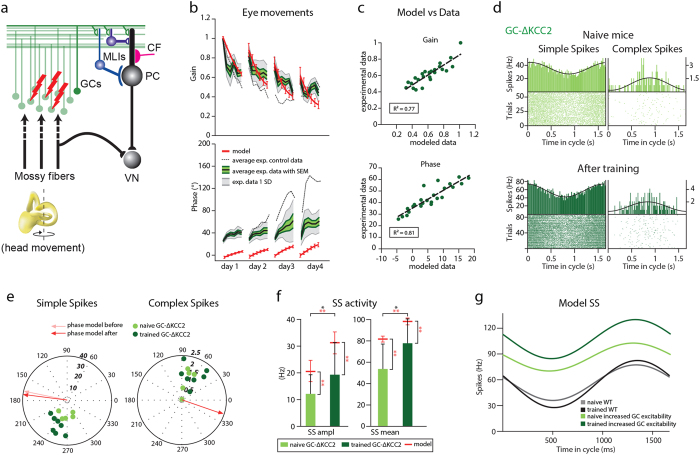
Predicted Purkinje cell activity before and after VOR phase-reversal is consistent with the experimental data from *GC-ΔKCC2* mice. (**a**) Part of cerebellar circuitry shown in Fig. 1a; Red lightning bolts indicate loss of KCC2 from GCs. (**b**) Experimental and modeled eye movements in *GC-ΔKCC2* mice as a function of training time of VOR phase-reversal training. Gain values (top panel) are normalized to the initial gain. Experimental data represent averages with SEM (green shaded area) and SD (grey shaded area) of the *GC-ΔKCC2* mice. Grey dotted line indicates the values of the littermate controls. Modeled changes are displayed for both gain and phase with SD (red line). (**c**) Linear regression plots displaying correlation between modeled and experimental data. (**d**) Representative PSTHs of floccular VA PC cells depict SS and CS modulation in *GC-ΔKCC2* mice before and after the VOR-reversal training during vestibular stimulation (0.6 Hz) (top and bottom panels, respectively). (**e**) Polar plots of SS (left) and CS (right) modulation before and after learning in *GC-ΔKCC2* mice (lighter and darker color, respectively) reveal increase in the modulation amplitude following learning. Each dot represents a single cell. Phase of the modeled SS and CS are indicated with the arrows. (**f**) As predicted by the model SS modulation in *GC-ΔKCC2* mice was much lower than that of the wild type before learning and increased after learning. Error bars denote SD; *denote p < 0.05; **denote p < 0.001. (**g**) SS activity displayed as a function of time in the cycle in the model with increased GC excitability (light green, initial value before learning; dark green, after training) and in controls (grey, initial value before learning; black, after training). The model predicts both the lowered modulation in naïve *GC-ΔKCC2* mice and an increase in the modulation following the training.

Cerebellar granule cells (GCs) are the most numerous neurons in the mammalian brain^37^ and many studies have shown that they are required to sustain a sufficient dynamic range of SS modulation and temporal variation^19^,^20^,^38^,^39^. We first investigated the spiking patterns following the VOR phase-reversal training in *GC*-Δ*KCC2* mice^11^,^20^, in which the potassium chloride co-transporter (Kcc2) is removed selectively from cerebellar GCs using the Cre-lox system with the Alpha6-promoter^26^ (Fig. 3a). This manipulation at the input stage results in a lowered spiking threshold^20^, which in turn leads to an increase in the intrinsic excitability of the GCs^40^, causing severe impairment of VOR phase adaptation^11^,^20^ (Fig. 3b and Supplementary Tables 4 and 5). When we quantified the predicted and experimental behavioral data of the *GC*-Δ*KCC2* mice, the linear regression revealed a strong correlation for the entire training for both gain (R^2^ = 0.77) and phase values (R^2^ = 0.81) (Fig. 3c and Supplementary Table 6). The day-by-day analysis of the VOR training showed that the predictive power of the model was high for days 1, 2 and 3 for gain values and for all days when it comes to predicting the phase (day 1, R^2^ = 0.60 gain and 0.81 phase; day 2, R^2^ = 0.78 gain and 0.89 phase; day 3, R^2^ = 0.66 gain and 0.95 phase; day 4, R^2^ = 0.15 gain and 0.60 phase). The average distance between the modeled and experimental values, as estimated with the use of least-square distance, was 0.09 for gain and 38.5**°** for phase.

We then proceeded to perform electrophysiological recordings *in vivo* from naïve and trained *GC*-Δ*KCC2* mice. Consistent with the behavioral findings, following the training the phase of the eye movements of the *GC*-Δ*KCC2* mice remained significantly lower than that of the control mice (p < 0.001, 53 ± 2° and 125 ± 7°, respectively, Tables 2 and 3). When we analyzed PC activity, we found that in naïve *GC*-Δ*KCC2* mice (n = 2, n of PCs = 5) the amplitude of SS was lower when compared to that in wild type mice (Fig. 3d) (peak-to-peak amplitude was 12.2 ± 3.2 Hz for *GC*-Δ*KCC2* mice and 32.2 ± 7.2 Hz in control mice; p = 0.04, Tables 1 and 4). The firing frequency in naïve *GC*-Δ*KCC2* mice was within a normal range, but increased significantly (p = 0.04) from 53.6 ± 10.5 Hz before training to 77.8 ± 7.6 Hz after the VOR adaptation (n = 2, n of PCs = 9). Following VOR adaptation SS modulation amplitude increased to 19.4 ± 4 Hz (p = 0.04) (Fig. 3e,f). Consistent with the model there was no significant shift in the phase of the SS modulation with respect to the table in the trained *GC*-Δ*KCC2* mice (265 ± 15**°** before and 244 ± 6**°** after; p = 0.08). As shown before, the model with increased granule cell excitability was able to learn the gain-decrease properly, yet could not consolidate during the dark and hence not learn the phase-reversal training^11^,^20^. When we increased granule cell excitability in our model, it predicted decreased SS modulation amplitude in the mutant mice and increased SS firing frequency and modulation following the training (Fig. 3f,g). Moreover, the model predicted that the increase in average SS firing rate following learning could be attributed to the bias towards potentiation in those mutant mice. Notably, even though the model and *in vivo* recordings of SS activity predicted the same direction of changes, the average values differed significantly from each other (p < 0.001). This is not surprising given the relatively small PC sample and variability of PC population coding.

### Disrupted simple-spike modulation due to loss of MLI inhibition

**Figure 4:**
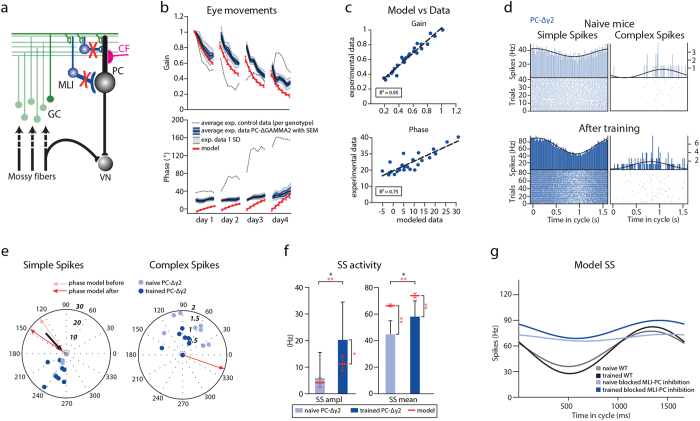
Experimental and modeled Purkinje cell modulation is disrupted in the *PC-Δγ2* mice. (**a**) Part of cerebellar circuitry shown in Fig. 1a; Red “X” depicts severed connectivity between MLIs and PCs in the *PC-Δγ2* mice. (**b**) Experimental and modeled eye movements in *PC-Δγ2* mice as a function of training time of VOR phase-reversal training. Gain values (top panel) are normalized to the initial gain. Experimental data represent averages with SEM (blue shaded area) and SD (grey shaded area) of the *PC-Δγ2* mice. Grey dotted line indicates the values of the littermate controls. Modeled changes are displayed for both gain and phase with SD (red line). (**c**) Linear regression plots displaying correlation between modeled and experimental data. (**d**) Representative PSTHs from *in vivo* recording in *PC-Δγ2* during vestibular stimulation (0.6 Hz). (**e**) Polar plots of SS and CS modulation before and after learning (lighter and darker blue, respectively). The plots reveal much lower modulation amplitude than that of the controls. Note that some PCs did not modulate their SS activity (indicated with black arrows). Each dot represents a single cell. Phase of the modeled SS and CS are indicated with the arrows. (**f**) Modeled and experimentally measured modulation was initially lower than that of the controls and increased significantly following learning in the *PC-Δγ2.* The firing frequency was also significantly higher following learning both in experimental and modeled data. Error bars denote SD; *denote p < 0.05; **denote p < 0.001. (**g**) SS activity as a function of time in the cycle in a control (grey, initial value before learning; black, after training) and in the model with blocked MLI to PC inhibition (light blue, initial value before learning; dark blue, after training).

To test how synaptic inhibition of PCs by MLIs influences the PC spiking patterns during learning, we recorded SS and CS activity before and after VOR phase-reversal training in two different cell-specific mutant mice in which the inhibitory input from MLIs onto PCs is affected. First we focused on the *PC*-*Δγ2* mice, in which the γ2-subunit of the GABAA receptor was deleted selectively from PCs using the Cre-lox system with the L7-promoter, specific for PCs^16^ (Fig. 4a). As mentioned above, this mutation leads to disruption of synaptic inhibition between MLIs and PCs and causes severe impairment of VOR adaptation^11^,^16^ (Fig. 4b and Supplementary Tables 4 and 5). When we quantified the predicted and experimental gain and phase values of the *PC*-*Δγ2* mice the linear regression revealed an almost perfect linear correlation for the entire training for gain (R^2^ = 0.95) and a strong correlation for phase values (R^2^ = 0.75) (Fig. 4c and Supplementary Table 6). The day-by-day analysis of the VOR training showed that the predictive power of the model was high for virtually all days of training for both gain and phase values (day 1, R^2^ = 0.96 gain and 0.46 phase; day 2, R^2^ = 0.99 gain and 0.74 phase; day 3, R^2^ = 0.96 gain and 0.93 phase; day 4, R^2^ = 0.78 gain and 0.93 phase). The average distance between the modeled and experimental values, estimated using least-square distance, was 0.12 for gain and 16.7**°** for phase.

In accordance with the behavioral data, the phase of the eye movements accompanying the electrophysiological recordings following training remained significantly lower in the *PC*-*Δγ2* mice than in control mice (p < 0.001, 66 ± 5° and 141 ± 7°, respectively, Tables 2 and 3). We found that in naïve *PC*-Δγ*2* mice (n = 3, n of PCs = 8) the amplitude of SSs was very low (peak-to-peak amplitude = 5.8 ± 3.4 Hz in *PC*-Δγ*2*) (Fig. 4d–f and Table 4) when compared to wild type mice (Fig. 2). In fact, in the *PC*-Δγ*2* mice out of 8 PCs only 3 PCs showed a measurable modulation (>1 Hz peak-to-peak modulation), while in the other 5 cells the measured modulation was close to 0. Interestingly, when we looked at the PC SS firing patterns in the mutant mice that underwent VOR phase-reversal training, we saw a significant increase in the amplitude of SS modulation (n = 3; n of PCs = 10; peak-to-peak amplitude = 20.2 ± 4.5 Hz; p = 0.03 when compared to SS modulation before training) (Fig. 4f and Table 4), which was reflected in an overall increase in SS firing frequency (44.9 ± 3.6 Hz and 58.2 ± 3.7 Hz, before and after training, respectively; p = 0.01).

When we removed the feedforward inhibition onto PCs and decreased the average strength of GC to PC synapses, a compensatory mechanism that has been observed in the *PC*-Δγ2 mice^11^,^16^, the model reproduced the spiking phenotype of *PC*-Δγ2 mice in that both the amplitude of modulation and firing frequency were significantly increased after learning (Fig. 4f and Table 4). Thus, here we show that the modeled SS activity is in line with both the experimental changes between naïve and “trained” PCs within the *PC*-Δγ2 mutant mice population and when compared with PC activity recorded in the control mice (Fig. 4g).

Even though our model does not rely on the local temporal patterns of SS activity, we also quantified the coefficient of variation for adjacent intervals (CV2) in the recorded PCs and this value was in line with previously reported findings (Supplementary Table 7) in that the CV2 of the *PC*-Δγ2 mice was significantly lower (p = 0.006) than that of the wild type mice^16^.

**Figure 5:**
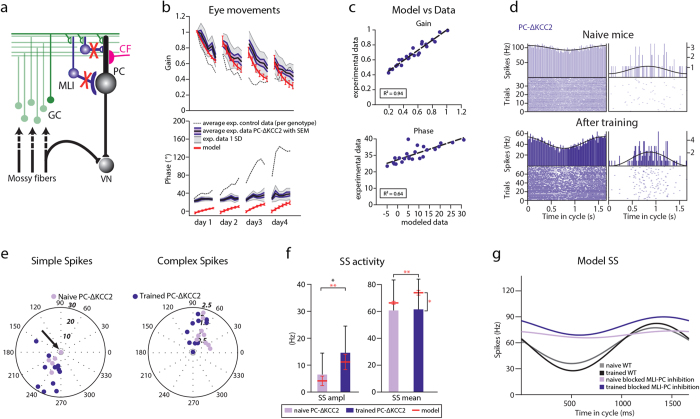
Experimental and modeled phase-reversal in the *PC-ΔKCC2* mice. (**a**) Part of cerebellar circuitry shown in Fig. 1a; Red “X” depicts severed connectivity between MLIs and PCs in the *PC-ΔKCC2* mice. (**b**) Experimental and modeled eye movements in *PC-ΔKCC2* mice as a function of training time of VOR phase-reversal training. Gain values (top panel) are normalized to the initial gain. Experimental data represent averages with SEM (purple shaded area) and SD (grey shaded area) the *PC -ΔKCC2* mice. Grey dotted line indicates the values of the littermate controls. Modeled changes are displayed for both gain and phase with SD (red line). (**c**) Linear regression plots displaying correlation between modeled and experimental data. (**d**) Representative PSTHs from *in vivo* recording in *PC -ΔKCC2* during vestibular stimulation (0.6 Hz). (**e**) Polar plots of SS and CS modulation before and after learning (lighter and darker purple, respectively). The plots reveal much lower modulation amplitude than that of the controls. Note that some PCs did not modulate their SS activity (indicated with black arrows). Each dot represents a single cell. Phase of the modeled SS and CS are indicated with the arrows. (**f**) Modeled and experimentally measured modulation was initially lower than that of the controls and increased significantly following learning in the *PC -ΔKCC2.* In contrast to the model, there was no significant increase to the firing frequency after training in *PC -ΔKCC2* mice. Error bars denote SD; *denote p < 0.05; **denote p < 0.001. (**g**) SS activity as a function of time in the cycle in a control (grey, initial value before learning; black, after training) and in the model with blocked MLI to PC inhibition (light purple, initial value before learning; dark blue, after training).

Next, we looked at behavioral and electrophysiological data from *PC*-Δ*KCC2* mice, in which the potassium chloride co-transporter (Kcc2) was deleted selectively from PCs using the Cre-lox system with the L7 promoter, specific for PCs^20^ (Fig. 5a). This mutation also leads to a significant disruption of synaptic inhibition between MLIs and PCs and also causes severe impairment of VOR adaptation^11^,^20^ (Fig. 5b and Supplementary Table 4 and 5). When we quantified the predicted and experimental gain and phase values of the *PC*-*ΔKCC2* mice, the linear regression revealed an almost perfect linear correlation for the entire training for gain (R^2^ = 0.94) and a strong correlation for phase values (R^2^ = 0.64) similar to that found for *PC*-Δγ2 (Fig. 5c and Supplementary Table 6). The day-by-day analysis of the VOR training showed that the predictive power of the model was high for virtually all days of training for gain values (day 1, R^2^ = 0.88; day 2, R^2^ = 0.93; day 3, R^2^ = 0.97; day 4, R^2^ = 0.94) and that the correlation for the phase values was high on days 1, 2 and 3 of the training (day 1, R^2^ = 0.68; day 2, R^2^ = 0.52; day 3, R^2^ = 0.71; day 4, R^2^ = 0.38). The average distance between the modeled and experimental values, estimated using least-square distance, was 0.18 for gain and 23.3**°** for phase.

Extracellular recordings in naïve *PC*-Δ*KCC2* mice (n = 3, n of PCs = 9) revealed very weak modulation of SS activity (Fig. 5d). Only 4 out of 9 cells modulated their SS in response to VOR stimulation (Fig. 5e). The modeled amplitude of SS modulation in naïve mice perfectly captured this impairment (peak-to-peak amplitude = 6.4 ± 2.7 Hz for experimental and 4.1 ± 0.3 for modeled data) (Fig. 5f and Table 4).

When we looked at the PC SS firing patterns in the mutant mice that were subjected to VOR phase-reversal training, we saw a significant increase in the amplitude of SS modulation similar to that found in the *PC*-Δγ2 (n = 3; n of PCs = 12; peak-to-peak amplitude = 14.5 ± 2.9 Hz; p = 0.05 when compared to SS modulation before training). The modeled amplitude of SS modulation in trained mice increased as well and was within one SD of the experimental data (Fig. 5f and Table 4).

Notably, the phase of the eye movements of the trained *PC*-Δ*KCC2* mice that were used for the PC recordings was indistinguishable from that of the naïve mice (36 ± 7° in naïve and 37 ± 4° in trained mice, Table 3) and significantly lower than that of the control mice (125 ± 7°, Table 2).

However, despite capturing the initial values of the SS firing frequency and induced changes in SS modulation following training, the model with blocked MLI to PC inhibition wrongly predicted an increase in SS firing frequency after the training in the *PC*-Δ*KCC2* mice. Experimentally, there was no observed increase in SS firing frequency following learning in the *PC*-Δ*KCC2* mice (p = 0.94), which may be due to the fact that the SS firing frequency of the *PC*-Δ*KCC2* mice was already higher in the naïve animals than in the *PC*-Δγ*2* mice (p = 0.04), leaving less room for an increase in firing frequency or SS modulation.

Finally, consistent with the fact that neither *PC*-Δγ2 nor *PC*-Δ*KCC2* mice are able to shift the phase of the eye movements despite the extensive training, we found no changes in the phase of SSs (p = 0.99, SS in *PC*-Δγ2; p = 0.48, SS in *PC*-Δ*KCC2,* Table 4), which was also predicted by the model.

### The model fails to capture phase-reversal in *PC*-*ΔPP2B* mutant

**Figure 6:**
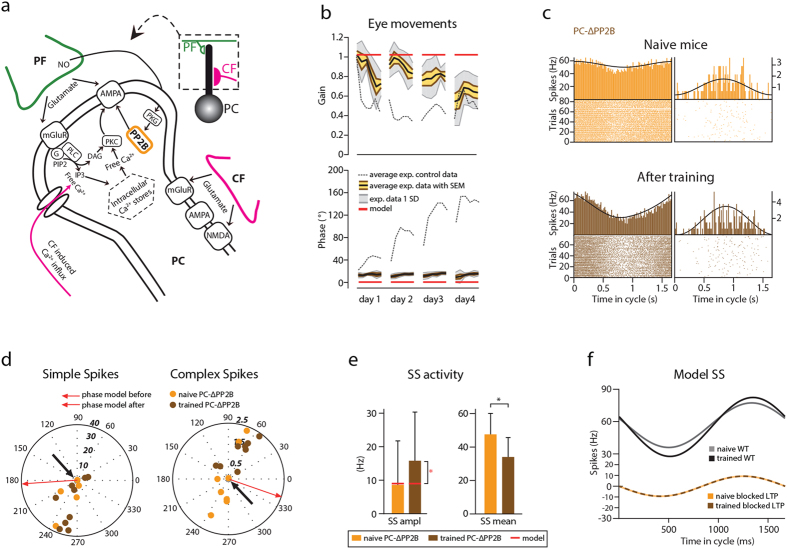
The model can reproduce neither behavioral nor electrophysiological changes during VOR phase-reversal in *PC-ΔPP2B* mice. (**a**) Simplified view of molecular pathways involved in PF to PC plasticity and role of protein phosphatase 2B (PP2B, orange frame). LTD is induced by simultaneous CF and PF activation (purple and green). A large Ca^2+^ transient resulting from Ca^2+^ influx together with release of Ca^2+^ from intracellular stores mediated by IP_3_, promotes protein kinase C (PKC) activation, which phosphorylates AMPA receptors leading to their internalization. PF activation also causes presynaptic release of nitric oxide (NO) and results in activation of protein kinase G (PKG), which inhibits PP2B and thus inhibits dephosphorylation of AMPA receptors. Stimulation of PFs alone leads to a much smaller Ca^2+^ influx, promoting activation of PP2B and regulating AMPA receptor insertion leading to LTP. (**b**) Experimental and modeled eye movements in *PC-ΔPP2B* mice as a function of training time of VOR phase-reversal training. Gain values (top panel) are normalized to the initial gain. Experimental data represent averages with SEM (orange shaded area) and SD (grey shaded area) of the *PC-ΔPP2B* mice. Grey dotted line indicates the values of the littermate controls. Note that the modeled values (red) are locked at the gain of 1 and phase of 0 due to the blockage of the potentiation at the PF-PC synapse. (**c**) Representative PSTHs from *in vivo* recording in *PC-ΔPP2B* during vestibular stimulation (0.6 Hz). (**d**) Polar plots of SS and CS modulation before and after learning (lighter and darker orange, respectively). Note that some PCs did not modulate their SS activity (indicated with black arrows). Each dot represents a single cell. Phase of the modeled SS and CS are indicated with the arrows. (**e**) Our model predicted low modulation of the SS modulation but failed to reproduce initial and trained firing frequencies. Error bars denote SD; *denote p < 0.05; **denote p < 0.001. (**f**) PC SS activity as a function of time in the cycle in a model with no PF-PC LTP. Note that the model cannot reproduce the experimental data due to the limitations explained in the discussion and methods.

We have previously shown that deletion of protein phosphatase 2B (PP2B, otherwise known as calcineurin) selectively from PCs in *PC*-Δ*PP2B* mice (Fig. 6a) results in loss of PF-PC long-term potentiation (LTP) and lowered intrinsic excitability of PCs^21^. In addition, deletion of PP2B in PCs causes disruption of motor performance, i.e. deficits in baseline of OKR, VVOR and VOR as well as motor learning^21^. In line with the experimental data, removal of LTP at the PF to PC synapse in the computational model severely affected learning (Fig. 6b and Supplementary Tables 4 and 5). However, it was impossible to quantify the goodness of fit between the model and experimental data, since the removal of the potentiation at the PF-PC synapse resulted in a “broken” output in which the gain and phase values are stuck at the gain of 1 and phase of 0° due to completely depressed weights at that synapse (Supplementary Fig. 2). When we remove LTP from our model, all the synapses become gradually depressed to their minimal value. Since the *PC*-Δ*PP2B* mice are not inducible knockouts, transcription of the PP2B2 protein is turned off early in development (around postnatal day 7 when Cre becomes expressed in PCs) and by the time the mice reach adulthood and begin training, all of their PF-PC synapses are already at the minimal values. Therefore, during the training, the synapses in our model can undergo neither LTP (due to lack of PP2B), nor LTD (because they are at the minimum).

We then investigated PC spiking patterns in *PC*-Δ*PP2B* mice before and after VOR phase adaptation training (naïve mice: n = 3, n of PCs = 9; trained mice: n = 6, n of PCs = 12). Consistent with the general behavioral findings in the *PC*-Δ*PP2B* mice, the phase of the eye movements of the *PC*-Δ*PP2B* mice that were subjected to electrophysiological recording following training remained at the same level as in naïve mice (26 ± 5° and 27 ± 4°, respectively, Table 3). Modulation amplitude of SSs in *PC*-Δ*PP2B* animals was low before the training and increased after VOR phase-reversal (8.6 ± 5 Hz before the training and 15.8 ± 4 Hz after; Fig. 6c–e, Table 4). However, due to large cell-to-cell variability this change was not significant (p = 0.27). Notably, firing frequency of SSs was significantly lowered following the training (47.4 ± 4 Hz before the training and 33.9 ± 3 Hz after; p = 0.02). Despite the fact that removal of the LTP “broke” the model, it was still able to correctly predict low initial amplitude of SS modulation (8.6 ± 5 Hz experimental and 9.3 ± 0 Hz modeled data) (Fig. 6e–f).

Interestingly, the phase of the CSs after training was significantly different from that before the training, due to the fact that some of the CSs before the training were modulated in phase with the SSs (Fig. 6d, left); the phase of CS modulation was 192 ± 33**°** before training and 75 ± 12**°** after (p = 0.004, Table 4). Since our model does not predict CS activity, we cannot make any computational predictions to the nature of this change (see Discussion for details).

### Silencing majority of granule cells prevents the model from learning VOR phase-reversal

**Figure 7:**
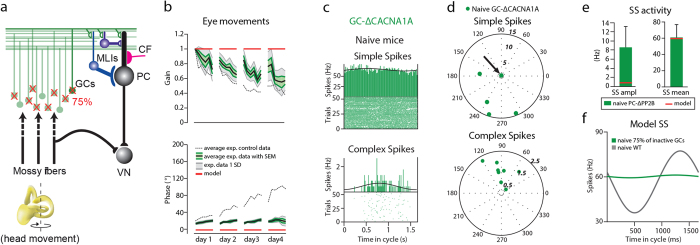
Silencing majority of granule cells prevents the model from learning the VOR phase-reversal. (**a**) Part of cerebellar circuitry shown in Fig. 1a; Red “X” symbols indicate loss of signal transmission from GCs. (**b**) Experimental and modeled eye movements in *GC-ΔCACNA1A* mice as a function of training time of VOR phase-reversal training. Gain values (top panel) are normalized to the initial gain. Experimental data represent averages with SEM (green shaded area) and SD (grey shaded area) of the *GC-ΔCACNA1A* mice. Grey dotted line indicates the values of the littermate controls. Note that the modeled values (red) are locked at the gain of 1 and phase of 0 due to the blockage of the potentiation at the PF-PC synapse. (**c**) Representative PSTHs from *in vivo* recording from naive *GC-ΔCACNA1A* mice, in which GC output is reduced by ~75% during VOR stimulation (0.6 Hz). (**d**) Polar plot of SS and CS responses to VOR stimulation in naïve *GC-ΔCACNA1A* mice. Each dot represents one cell. Phase of the modeled SS and CS are indicated with the arrows. (**e**) Modulation of SS, is attenuated in *GC-ΔCACNA1A* mice. Error bars denote SD. (**f**) In our model the effect of reducing GC output by 75% and blocking PF-PC long–term plasticity is in line with the experimental data, in that the modulation depth of simple-spikes in Purkinje cells of *GC-ΔCACNA1A* mice is also significantly reduced during VOR.

Given that our computational model was able to accurately predict the changes in cerebellar spiking patterns and motor learning impairment in the mutant mice with increased intrinsic excitability of granule cells, we next explored whether it was able to reproduce learning deficits when the output from the vast majority of granule cells was minimized. To that end we looked at the *GC*-*ΔCACNA1A* mutant mouse, in which the CaV2.1 (P/Q-type) Ca^2+^ channels necessary for neurotransmitter release at their parallel fiber terminals is selectively deleted from a subset of the GCs^19^. This mutation results in a reduction of GC to PC output by approximately 75% as well as in impaired long-term plasticity (both LTP and LTD) at GC-PC synapses (Fig. 7a). As a consequence of these disruptions these mice were unable to successfully complete the VOR phase-reversal paradigm (Fig. 7b). Implementing a 75% reduction in GC output and loss of PF-PC long-term plasticity in the computational model yielded similar results in that adaptation of the VOR was virtually absent.

However, similarly to the modeled *PC*-Δ*PP2B,* it was impossible to quantify the goodness of fit between the model and experimental data, since the impaired long-term plasticity at GC-PC synapses resulted again in a “broken” output, in which the gain and phase values are stuck at the gain of 1 and phase of 0° due to the fact that the weights cannot be adjusted in either direction (no LTP or LTD) (Supplementary Fig. 3a).

Interestingly, as previously described^19^,^41^, modulation of SS activity, during visual stimulation, was significantly reduced in *GC*-*ΔCACNA1A* mice compared to that of control littermates (littermate controls: n = 7, n of PC = 13; *GC*-*ΔCACNA1A*: n = 3, n of PC = 13; p = 0.001) (Supplementary Fig. 3b,c). In line with the selective effect of the mutation in the MF-GC-PF pathway, the modulation of CSs was not significantly altered (p = 0.7).

Here, we confirmed the lowered modulation of SS amplitude measuring PC activity during VOR in naïve *GC*-*ΔCACNA1A* mice (Fig. 7c). PCs (n = 9) showed very weak modulation (8.6 ± 1.6 Hz) and some cells did not modulate at all (Fig. 7d). The SS mean frequency was within the normal range (Fig. 7e and Table 4).

The *GC*-*ΔCACNA1A* model was still able to some extent to predict low initial amplitude of SS modulation (8.6 ± 1.6 Hz experimental and 0.9 ± 0 Hz modeled data) and intact SS firing frequency (60.5 ± 5.2 experimental and 60.1 ± 0 Hz modeled data) in naïve mice (Fig. 7f).

### The model can reproduce Purkinje cell activity and eye movements during VOR of mice with uncrossed climbing fibers

**Figure 8:**
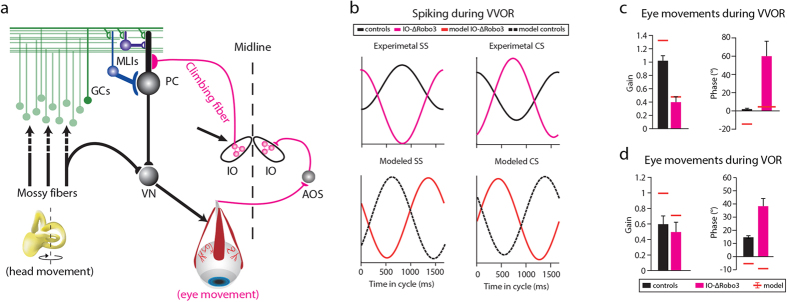
The model can account for 180 degree shift in the PC activity and eye movement impairment in *IO-ΔRobo3* mice during VOR. (**a**) Schematic illustration of the altered olivocerebellar circuitry in *IO-ΔRobo3* mice. For the wildtype schematics and abbreviations see Fig. 1a. Note that the climbing fibers (CF, purple) project to the PCs on the ipsilateral side. (**b, top**) Representative PSTHs of SS and CS activity from *in vivo* recording during VOR stimulation as a function of time in the cycle in *IO-ΔRobo3* (purple) and littermate controls (black). (**b, bottom**) SS and CS activity predicted by the model with ipsilateral projecting CFs (purple) and controls (black dashed line). Note that the peak of the modulation is reversed with respect to the wild type mice, which is consistent with experimental data (raw experimental data obtained from: Badura *et al.*^22^). (**c**) Gain and phase values of the eye movements during VOR (0.6 Hz) in naïve wild type (black) and *IO-ΔRobo3* (purple) mice. Error bars denote SD; *denote p < 0.05; **denote p < 0.001.

In a wild type mouse IO neurons send their axons, otherwise known as climbing fibers, to the contralateral cerebellum innervating the proximal dendrites of the Purkinje cells. It has been shown that deletion of the IO pathway leads to profound motor deficits^42^,^43^,^44^,^45^, but what is striking is that the most severe phenotype results from rerouting the climbing fibers so that they project to the ipsilateral cerebellum (Fig. 8a) 22. As a result all forms of plasticity in the molecular layer of the cerebellar cortex will be expressed with opposite effects in terms of directionality^2^. The *IO-ΔRobo3* mice have dramatic motor performance deficits and severe ataxia. In our previous work, we showed that this behavioral phenotype is accompanied by an almost 180 degree shift in both CS and SS modulation, maintaining the reciprocity of CS-SS firing during VOR (Fig. 8b, top). We therefore concluded that it is the CF input that shapes the phase of the SS activity of PCs^22^. To find out whether our model could capture the shifts in SS and CS modulation while maintaining the reciprocity, we shifted the phase of the CF input by 180 degrees. As a result, the phase of the SSs predicted by our model also reversed, maintaining the reciprocal modulation (Fig. 8b, bottom). Importantly, the model also predicted performance deficits in eye movements during baseline VOR in naïve *IO-ΔRobo3*, which is consistent with our previously published experimental findings (Fig. 8c and Table 5). Notably there were some significant differences between the modeled predictions and experimental data. First, the SS modulation amplitude was significantly lower in the modeled data (p < 0.001). Second, the predicted values for gain and phase of the eye movements were significantly lower for the uncrossed mutants than the ones observed experimentally (p < 0.003 for both gain and phase). These differences might result from the fact that the model does not include the influence of CF onto MLIs, which had a big effect on the MLI activity in the *IO-ΔRobo3* mice (see Discussion for details).

## Discussion

Understanding how neuronal activity relates to animal behavior has been an outstanding challenge in systems neuroscience. We now understand that, with the exception of primary sensory systems, there is no straightforward one-on-one relationship between the activity of a given neuron and behavior. Rather, the neuronal networks are dynamic systems where transitions from one state to the other are often non-linear. Therefore, we need good mechanistic models, which by conceptualizing a given system, can predict how changes at the level of one part of the network affect the rest of the assembly^46^,^47^.

In this paper we first carefully quantify the extent to which our model is predictive of behavioral impairments in learning the VOR reversal training in five mutant lines with cerebellar cortical deficits (*GC*-Δ*KCC2, PC*-*Δγ2, PC*-Δ*KCC2, PC*-Δ*PP2B* and *GC*-*ΔCACNA1A* mice). We combine these data with electrophysiological recordings of flocculo-nodular Purkinje cell SS and CS activity before and after VOR phase-reversal adaptation in multiple cerebellar specific knock-out mice with known behavioral deficits (*GC*-Δ*KCC2, PC*-Δ*PP2B, PC*-*Δγ2,* and *PC*-Δ*KCC2* mice). With the exception of the *PC*-Δ*PP2B* mutants, we observe that despite different pathways being affected by those mutations, whether it is GC excitability, PF-PC plasticity or MLI-PC inhibition, at the level of Purkinje cell activity, the outcome is a net reduction in depth of the SS modulation. These changes reflect what we modeled to be a reduction in weights at the GC to PC synapse, bringing them closer to a lower bound and giving PCs in mutant mice less room to adjust their weights during learning. Likewise, we showed in a mutant, in which the inferior olive in the ventral lower brainstem is affected (i.e. the *IO-ΔRobo3*), that the phase of the SS modulation can be predicted by the phase of the climbing fiber activity that arises in this nucleus. Together these findings suggest that even minor shifts in activity can be predictive of deficits in learning when put in the framework of a well-designed mechanistic model.

To obtain insight into the working mechanisms of learning a reflex like the VOR we can benefit substantially from both the qualitative and quantitative experimental verifications and falsifications of our computational predictions. Interestingly, some of the SS firing characteristics of *PC*-*Δγ2* and *PC*-Δ*KCC2* mice varied, even though both mutations result in loss of phasic inhibition at the MLI-PC synapse. Those differences may be explained by several factors. First, the nature of the mutation could trigger different compensatory mechanisms, some of which might not have been measured in the original studies. Second, due to large variability in PC spiking, which is consistent with the idea of widely distributed population-coding^48^, our study might be under-sampling the PC population.

In addition, we show that our model is robust enough to predict SS activity in naïve *GC*-*ΔCACNA1A* mice and dramatic motor performance deficits in *IO*-*ΔROBO3* mice. In *GC*-*ΔCACNA1A* mice, the cell-specific deletion of the CACNA1A gene minimizes the output of cerebellar granule cells and disrupts PF-PC plasticity (both LTP and LTD). As could be expected, the simple spike modulation depth diminished during visually induced modulation, which typically results in even deeper modulation than during vestibular stimulation^49^. This virtual absence of modulation *in vivo* was captured by our model, as it predicted an attenuated modulation depth during VOR. Despite the fact that the removal of LTP at the PF-PC synapse “broke” the model in the case of *PC*-Δ*PP2B* mice, in the case of *GC*-*ΔCACNA1A* mice the model could recapture the baseline SS firing frequency and amplitude. This was due to the fact that simultaneous impairment of LTP and LTD at the PF-PC synapse, as was shown for the *GC*-*ΔCACNA1A* mice, leaves intrinsic firing of PCs intact (at around 60 Hz). Interestingly, *GC*-*ΔCACNA1A* mice did show a slow decrease in VOR gain over days, a feature that was not captured by the model. This finding hints towards the presence of alternative mechanisms or sites of plasticity downstream in the vestibular nuclei.

Rerouting the climbing fibers in the *IO*-*ΔROBO3* mice so that they project to the ipsi- rather than contralateral cerebellum leads to an almost 180-degree shift in CS and SS modulation both in the modeled and experimentally tested PCs. The model suggests that, due to the fact that CF shapes learning at the GC to PC synapses, a 180-degrees shift in the CSs automatically imposes a 180-degrees shift of the SSs. We therefore conclude that our model of VOR phase-reversal adaptation provides a plausible explanation for the behavioral impairments. The increased depth of SS modulation in the *IO-ΔRobo3* mice, with respect to their littermate controls, is at least to a large extent a result of shifted MLI modulation^22^. Since our model does not include plasticity in the MLIs or direct CF to MLI input this shift in their activity cannot occur and therefore the predicted depth of SS modulation is at odds with that measured experimentally.

One of the caveats of working with cell-specific knock-out mice, which are not conditional mutants, is that the network has sufficient time to adjust to the loss of a certain pathway or disruption of synaptic and/or intrinsic plasticity. Given that the brain is highly plastic, especially throughout development, there are multiple compensatory mechanisms, which may help to cope with the loss of a certain gene or protein^50^,^51^. We show that our model can cope with most of them. In the future it would be interesting to investigate how acute changes to the cerebellar circuitry affect the dynamics of the physical and modeled cerebellar network.

Notably, our model failed to reproduce electrophysiological data collected from *PC*-Δ*PP2B* mice, in which LTP and intrinsic plasticity are selectively abolished in PCs. When we removed LTP in our model the PF-PC synapses were only undergoing depression, eventually bringing the weights to their lower bound. As we can see from the experimental results, this is clearly not the case in the *PC*-Δ*PP2B* mutant, which in fact may provide an interesting clue. Probably, we need to include plasticity at the level of the GC-MLI and/or MLI-PC synapse^2^. Indeed, the model only first learns through the modification of the GC to PC synapses, which can then be transferred to the MF to MVN synapses. The model does not take into account plasticity at other synapses, such as in the molecular interneurons, limiting the learning possibilities and missing the actual opportunity of the *PC*-Δ*PP2B* mutants to normalize SS modulation via MLI plasticity. But if *PC*-Δ*PP2B* mice show a relatively normal SS modulation, why then do they not show gain-increase or phase-reversal learning? A possibility is that PP2B may also be required for presynaptic plasticity and/or synaptic transmission at the level of the PC axon terminals^52^, thus in effect minimizing the downstream impact of the increase in SS modulation that still occurs through MLI modulation and plasticity. It might also explain why gain-decrease modulation can, to some extent, still take place in the *PC*-Δ*PP2B* mice^21^, as this process may largely depend on the MF to MVN interaction^41^. Finally, it should be noted that we also did not implement any form of homeostatic control mechanism, such as synaptic scaling, into our model^53^. As a consequence, in our model, the GC to PC synapses of the *PC*-Δ*PP2B* mice only undergo LTD, driving the GC to PC synapses to their lower bound due to continuing CF activity. In actual slice experiments of *PC*-Δ*PP2B* mice, GC to PC synapses are not stuck at their lower bound, as they can still undergo LTD^21^ and thereby possibly still contribute to changes in SS modulation. In the future, the model will be further refined by taking into account homeostasis and learning in molecular interneurons, such that it reproduces these experimental data. Thus, one may consider the limitations described above as one of the strengths of our model, in that it can be used as a screening method to identify likely neuronal components, which are the targets of a short- and long-term compensatory mechanisms^54^.

Given that a substantial part of the cerebellar research community involved in eye movement studies uses gain increase and decrease training paradigms, it would be of interest to know if our model can predict and accurately depict all training routines. Gain decrease is part of the VOR reversal learning and is incorporated into our model. Therefore, we are confident the model could be of use to our peers who are interested in this type of VOR adaptation. Although we have not extensively tested gain increase training in our model, initial tests suggest that it can explain the increase in gain of the eye movements during gain up training. Furthermore, we speculate that the *GC*-Δ*KCC2,* as well as the *PC*-*Δγ2* and *PC*-Δ*KCC2* mutant models will not be impaired in their gain increase. Note that they all have a successful gain decrease, but they are impaired for their phase reversal. On the other hand, we speculate that the *PC*-Δ*PP2B* and *GC*-*ΔCACNA1A* mutant models would not undergo gain increase, as these models do not exhibit any learning in general. We would like to encourage the cerebellar research community to explore our model to test its predictions in many paradigms revealing its strengths and limits. Indeed, as many computational scientists argue, the analysis of models that turn out to be in conflict with observations often gives more insight about the system than a model whose predictions are roughly in line with observations^55^.

## Materials and Methods

All experiments involving transgenic mice were approved by the animal welfare committee (Erasmus MC, Rotterdam, The Netherlands) and conducted in accordance with European and Dutch guidelines and legislation. All mice used in our studies were adult males.

### Mouse lines

#### GC-ΔKCC2 mice

*Δα6::Cre;Kcc2*^*lox/lox*^ mice were described previously^11^,^20^. In short, the GC-specific ablation of the potassium chloride cotransporter (Kcc2) was achieved by crossing *Kcc2*^lox/lox^ mice^20^ with *Δα6::Cre* mice^31^. This resulted in increased excitability of the GCs (by lowering their spiking threshold). Adult (10–16 week old) male *Δα6::Cre*;*Kcc2*^*lox/lox*^ mice, referred to as *GC*-ΔKCC2 mutants (n = 4) were used for experiments.

#### PC-Δγ2 mice

Generation of *γ2I77*^*lox/lox*^, *L7/Pcp2::Cre* and *L7/Pcp2::Cre*;γ2I77^*lox/lox*^, mouse lines was described previously^11^,^16^,^56^. In short, the PC-specific ablation of the *γ2*-subunit of the GABAA receptor, which is required for targeting the receptor to the postsynaptic membrane, was achieved by crossing *γ2I77l*^lox/lox^ and *L7/Pcp2::Cre* mice^16^. This resulted in loss of GABAergic transmission from MLIs to PCs. Adult (10–16 week old) male *L7/Pcp2::Cre*;γ2I77^*lox/lox*^ mice, referred to as *PC*-Δγ2 mutants (n = 6) were used for experiments.

#### PC-ΔKCC2 mice

*L7/Pcp2::Cre;Kcc2*^*lox/lox*^ mice were described previously^11^,^20^. In short, the PC-specific ablation of the potassium chloride co-transporter (Kcc2) was achieved by crossing *Kcc2*^lox/lox^ mice with *L7/Pcp2::Cre* mice^20^,^56^. This resulted in strong reduction of GABA-induced hyperpolarization of PCs, effectively removing the inhibition from MLIs on PCs. Adult (10–16 week old) male *L7/Pcp2::Cre*;*Kcc2*^*lox/lox*^ mice, referred to as *PC*-ΔKCC2 mutants (n = 6) were used for experiments.

#### PC-ΔPP2B mice

Mutant mice in which the regulatory subunit (CNB1) of calcium/calmodulin-activated protein phosphatase 2B (PP2B, otherwise known as calcineurin) was selectively deleted from PCs were described previously^21^. This ablation resulted in loss of PF-PC long term potentiation (LTP) and lowered intrinsic excitability of PCs. In this study adult (10–16 week old) male *L7/Pcp2::Cre*;*PP2B*^*lox/lox*^ mice, (n = 9) were used for experiments.

#### GC-ΔCACNA1A mice

In short, this mutation results in granule-cell-specific knockout of P/Q-type voltage-gated calcium channels (VGCCs), which normally mediate ~90% of neurotransmitter release from GC axons. This mutation effectively leads to silencing the output of ~75% of the granule cells (for details see in Galliano *et al.*^19^). In this study adult (10–16 week old) male *Δα6::Cre*;*Cacna1a*^*lox/lox*^ mice, referred to as *GC*-*ΔCACNA1A* mutants (n = 9), were used for experiments for electrophysiological recordings during VOR baseline.

#### IO-ΔRobo3 mice

All experimental data for the *Ptf1a::cre;Robo3*^*lox/lox*^ mice were published before^22^. In short, this mutation results in an inferior olive (IO) specific deletion of the Robo3 gene, which in turn leads to a complete failure of IO axons to cross the midline. This effectively means that in the *Ptf1a::cre;Robo3*^*lox/lox*^ mice climbing fibers are rerouted so that they project only to the ipsilateral side. No new mice were used for this study.

### Eye movement recordings

Baseline eye movements and VOR adaptation training were recorded as previously described^11^,^16^,^20^,^21^. In short, mice were headplated under general anesthesia with isoflurane/O^2^. After 3 days of recovery, mice were head-restrained with the headplate fixed to a metal bar for 1 h habituation session. The restrainer was fixed onto a turntable, surrounded by a cylindrical screen with a random-dotted pattern. Eye movements [optokinetic reflex (OKR), visual VOR in the light (VVOR) and VOR] were evoked respectively by rotating the screen, the screen and the turntable or the turntable alone at different frequencies. The positions of the table and drum were recorded by potentiometers. Eye movements were recorded, as previously described^57^,^58^, with the use of an infrared CCD camera fixed to the turntable. At the beginning of each session the eye movement calibrations were computed as previously described^59^,^60^. Mice were submitted to baseline measurements and VOR phase-reversal training for 5 consecutive days (1–1.5 h long sessions). Phase-reversal paradigm: Day 1, in-phase stimulation of drum and turntable with fixed amplitude (5 × 10 min periods of sinusoidal in phase drum and table rotation at 0.6 Hz, both with an amplitude of 5°) aimed at reducing the gain of the VOR (this day was not modeled since it did not contain any mismatch in the phase between the stimuli); Days 2, 3, 4, and 5, in-phase stimulation of drum and turntable with increasing amplitude of the drum rotation and fixed amplitude of the turntable [5 × 10 min periods of sinusoidal rotation at 0.6 Hz, but with drum amplitudes of 7.5° (day 2) and 10° (days 3, 4, and 5), while the amplitude of the turntable remained 5°; Fig. 1b), aimed at reversing the phase of the VOR. Gain and phase values of the VOR eye movements were measured in the dark after each 10 min training session by rotating the turntable (frequency 0.6 Hz, amplitude 5°) and calculated offline using custom-made Matlab routines (The MathWorks, Natick, MA, USA)^58^. The animals were kept and transported in and out of the setup in the dark in between all recording days. After the 5th day of VOR phase-reversal training, mice were deeply anesthetized and a craniotomy was made in the left occipital bone to allow for electrophysiological measurements (for details see section on *in vivo* electrophysiology below). All raw behavioral data of phase-reversal experiments were obtained from previously published papers. However, for the purpose of this paper we have re-analyzed the data using vector averaging method^61^, allowing more accurate quantification.

### *In vivo* electrophysiology

Single unit recordings of floccular PC activity responding to the vertical axis stimulation (VA cells) in awake mice exposed to the vestibular stimulation were performed as described previously^11^,^22^. In short, naïve mice were put under general anesthesia and headplated. Following that procedure a craniotomy was made in the left occipital bone (without damaging the dura) and an acrylic cement chamber was built around the craniotomy; the chamber was sealed with bone wax. All mice received an analgesic treatment after the surgery (temgesic/buprenophine subcutaneous injection 0.015 mg/kg). After 3 days of recovery, mice were submitted to experimental procedures (electrophysiological recordings). Mice that underwent the VOR phase-reversal training had a pedestal used for the head fixation and hence the surgery was restricted to placing the craniotomy in the occipital bone. It should be noted that during the relocation to and from the operating room, where the craniotomy was made, trained mice were anesthetized in the dark and their eyes covered with a thick layer of Duratears (before the transition). With the exception of the exposure to short optokinetic stimulation during the recording sessions, trained animals were kept and transported in and out of the setup in the dark during the experiments to prevent loss of the acquired phase adaptation. Naïve animals received one training session (1 h in the restrainer) before experiments to habituate to the experimental settings, but since the trained animals were accustomed to the setup they were used directly for electrophysiological recordings without additional habituation. During the recording sessions animals were placed in the restrainer fixed onto the turntable with a cylindrical screen with a random-dotted pattern surrounding the turntable. The turntable was equipped with an electrode manipulator, which guided the borosilicate glass electrodes into the brain. Single unit, extracellular signals were recorded in awake mice, from the floccular PCs, identified by their CS responses. Single units were confirmed by short pause in SS firing following each CS (CF pause)^62^. Only cells that responded optimally to stimulation around the vertical axis were used in this study. Short optokinetic stimulation (<60 s) was used to identify the VA PCs. Activity of the positively identified VA PCs was subsequently recorded during vestibular stimulation by rotating the turntable in the dark at frequency of 0.6 Hz and amplitude of 5 degrees. We have excluded cells that did not meet the following criteria: 1) single unit isolation for at least 60 s; 2) stable baseline (no drift); 3) stable size of CS an SS from one table cycle to another. Signals were filtered, amplified and stored for the off-line analysis. After the experiments mice were euthanized by cervical dislocation under isoflurane anesthesia.

### Model

The model is a mathematical implementation of Fig. 1a and has been developed as presented in Clopath *et al.*^11^. The MFs are encoding the head velocity. They project onto the GCs in the granule layer, which then relay the signal onto PCs at the PF to PC synapse as well as onto the MLIs. In our model each granule cell fires at a different phase in the cycle together covering the entire sinusoidal stimulation. The PF to PC synapse is plastic and therefore can be potentiated and depressed which would lead to an increase or decrease in the PC output, the VN. The VN also receives direct projections from MFs, these projections are plastic, and the VN’s output in return drives the eye movements. The PCs also receive two additional inputs, one inhibitory form the MLIs and one powerful excitatory projection from the IO through the CF. The CFs activity carry an error signal of the retinal slip and modulate the PF to PC plasticity. In our model the learning initially occurs at the PF to PC synapse and is then gradually transferred onto MF to VN synapse.

All the parameters of the model were taken from Clopath *et al.* and were kept fixed in this study. The only alterations were introduced to recapture the changes in the circuitry seen in the mutants (see the details below).

### Dynamics of activity variables

The mossy fibers encode the head velocity^63^ according to the equation


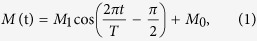


where M_1_ = 1/4, M_0_ = 1/4 and T = 1666 ms is the period of the rotation of the turntable (0.6 Hz).

The granule cell network is composed of N = 100 granule cells, whose activity is driven by the mossy fibers, but with a different phase shift for each cell. We also performed simulations with a larger number of granule cells (N = 1666) with no noticeable difference. The distribution of phase shifts is such that there is a bias towards the phase of the MF inputs^64^. The activity of granule cell i, G_i_(t) can be written as


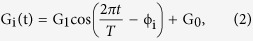


for i = 1..N, where G_1_ = 1 (for the wild-type mice), G_0_ = 1 and


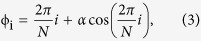


where α = 0.19.

The activity of the molecular layer interneuron network is described by a single variable I(t), which is proportional to the average activity in the granule cell network


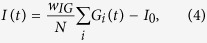


where I_0_ = w_IG_ G_0_ − 0.85 is an inhibitory term and w_IG_ = 2.5 measures the strength of the synaptic weight from granule cells. As a result of this parameter choice, I(t) is more modulated in phase with ipsiversive head movements than GC activity. This assumption is critical to reproduce the modulation profile of Simple-spikes of the PCs.

The activity of the PC network depends on both, direct excitation from GCs, and feedforward inhibition from interneurons, i.e.


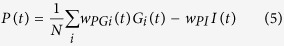


where w_PGi_ are the weights from granule cell G_i_ to PC and w_PI_ = 1 (in the case of the wild-type) is the weight from IN to PC.

The activity in MVN is described by two variables V_E_ and V_I_, representing the excitatory and inhibitory populations in that structure^65^. Both variables depend on both the excitatory input from MF and the inhibitory input from PC, i.e.


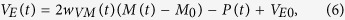



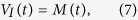


where w_VM_(t), are the excitatory weights from MF to excitatory/inhibitory MVN populations, and V_EO_ = 2.25. The motor command is assumed to be proportional to the difference between V_E_(t) and V_I_(t), V(t) = V_E_(t) − V_I_(t), since both excitatory and inhibitory neurons project to oculomotor motor neurons^65^.

The target motor command V_t_ is defined as


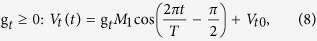



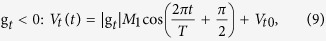


where g_t_ is the target gain and V_t0_ = 1.

The climbing fiber activity C(t) is assumed to be weakly modulated by head movement in the dark^30^ and, when light is present, by contraversive retinal slip (the ‘error signal’), V(t − δ) − V_t_(t − δ) where delta is the delay in this error signal. C(t) can be written as


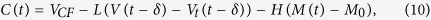


where L = 1, 0 in light/dark conditions, ν_CF_ is the baseline firing rate of C, and H = 0.03 is the modulation by head movement. The assumption that C is modulated in phase with the head movement is important in order to reproduce the temporal modulation of Complex-spikes of the PCs.

### Dynamics of synaptic weights

There are two learning sites, one at the GC to PC synapses and one at the MF to V_E_ synapses. The plasticity at the GC to PC synapse is described by the following expression,


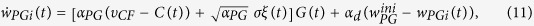


where ξ is white noise with zero mean and unit variance density, σ = 0.02 is the amplitude of the noise, and α_PG_ = 3.5 × 10^−5^ ms^−1^ is the learning rate. All synaptic weights have an upper bound at 2.85 and a lower bound at 0.85, consistent with experimental data on LTP/LTD, showing a limited range of synaptic efficacies^66^,^67^. Finally, the weights slowly decay to their initial value w_PG_^ini^ = 1.85 with a slow decay rate α_d_ = 4.5 × 10^−6^ ms^−1^. The weight update is in good agreement with the plasticity seen experimentally at the GC to PC synapses, i.e. potentiation under GC stimulation and depression under CF and GC co-stimulation^2^,^6^,^68^.

The synaptic weight from MF to V_E_, w_VM_, is decreased when MF and PC are co-active or co-inactive and increased if one of the two is active. This plasticity was observed experimentally in ref. 69. It can be written as


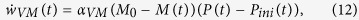


α_VM_ = 5.6 × 10^−6^ ms^−1^ is the learning rate and P_ini_(t) is P(t) before training. There is a hard lower bound at 0. The weight is initialized to w_VM_^ini^ = 0.88 so that V produces a gain of 1. Indeed, since PC is initially modulated with the head movement already, w_VM_^ini^ needs to be smaller than 1, to obtain a gain of 1. In the model, plasticity is present all the time, irrespectively of whether it is dark or light.

### Model adapted for the mutant mice

In the case of the first granule cell mutant (*GC-*Δ*KCC2*)^20^, we increase the excitability of GC and therefore set G_0_ = 1.8. In order to have the same P_ini_ as the wild-type, we set w_PG_^ini^ = 1.85, and for V to have an initial gain of 1, we set w_VM_^ini^ = 0.7.

In the case of the second granule cell mutant (*GC*-*ΔCACNA1A*)^19^ we have removed 75% of the GCs and disabled LTP and LTD at the GC to PC synapse.

In the case of the *PC*-*ΔPP2B* mice^21^, we blocked LTP from the GC to PC synapse.

In the case of the inhibitory knock-out model (*PC*-*Δγ2* and *PC-ΔKCC2*)^16^,^20^, we removed the inhibition onto PC and therefore set w_PI_ = 0. In order to have the same P_ini_ as the wild-type, we set w_PG_^ini^ = 1, and for V to have an initial gain of 1, we set w_VM_^ini^ = 1.19.

In the case of CF-uncrossed mice^22^, we shifted the CF by 180-degrees.

### Simulation protocol of the model and parameter setting

The model was used to reproduce the phase-reversal learning task^16^. The table rotates at 0.6 Hz. Before the learning protocol, an initialization phase is performed: the model is simulated for 50 cycles with a target gain of 1, g_t_ = 1, followed by two nights in the dark, i.e. 2880 cycles. Then the phase-reversal-learning task starts. For the first 50 cycles, the target gain is set to g_t_ = 0 (day 1 training), then 1440 cycles with no retinal slip (corresponding to the first night), then 50 cycles at g_t_ = −0.5 (day 2), then 1440 cycles without retinal slip (night 2), then 50 cycles at g_t_ = −1 (day 3), then 1440 without retinal slip (night 3), then 50 cycles at g_t_ = −1 (day 4), then 5*1440 cycles without retinal slip (corresponding to 5 days where the animals are kept in the dark). In the numerical simulations, equations are integrated with a time = step of dt = 1 ms. Weight changes are updated in a batch manner at the end of every cycle. Every simulation is repeated 30 times in order to calculate the mean and the error bars which indicate 1 standard deviation.

### Normalization of the PC spiking output

In order to make a comparison between electrophysiological data and predictions produced by the model, the SS output was normalized to the mean FF frequency of the control mice (37 cells). The selected normalization factor of 60.05 Hz was kept constant for all mutants and controls.

### Data analysis

Eye movement recordings were analyzed using custom made Matlab routines Matlab (Mathworks, MA, USA)^61^,^70^. Electrophysiological recordings were analyzed using SpikeTrain (Neurasmus B.V., The Netherlands, www.neurasmus.com), running under Matlab (Mathworks, MA, USA). Modulation amplitude of SS activity was calculated for both the model and experimental data as the peak of the SS activity (in Hz) minus the trough of the SS activity (in Hz). For quantitative analysis between the model and experimental data we used linear regression analysis, least-square distance regression to quantify the goodness of fit for the eye movement data, and two-tailed t-test for the electrophysiological data. Unless specified otherwise, the ± indicate SEM.

## Additional Information

**How to cite this article**: Badura, A. *et al.* Modeled changes of cerebellar activity in mutant mice are predictive of their learning impairments. *Sci. Rep.* **6**, 36131; doi: 10.1038/srep36131 (2016).

**Publisher’s note:** Springer Nature remains neutral with regard to jurisdictional claims in published maps and institutional affiliations.

## Supplementary Material

Supplementary Information

## Figures and Tables

**Table 1:** Purkinje cell activity during electrophysiological recordings before and after phase reversal adaptation in all control mice.

	SS naïve	SS after	CS naïve	CS after
***Controls C57Bl/6***	**n = 14**	**n = 12**	**n = 14**	**n = 12**
FF (Hz)	58.29 ± 3.09	70.61 ± 4.31	0.97 ± 0.07	0.92 ± 0.06
AMPLITUDE (Hz)	20.86 ± 2.07	27.64 ± 1.32	0.87 ± 0.08	0.51 ± 0.07
PHASE	249 ± 16	285 ± 10	115 ± 23	83 ± 12
***Controls ΔKCC2***	**n = 10**	**n = 7**	**n = 10**	**n = 7**
FF (Hz)	63.45 ± 4.89	75.72 ± 3.04	0.99 ± 0.06	0.84 ± 0.11
AMPLITUDE (Hz)	32.26 ± 7.20	57.64 ± 11.1	1.56 ± 0.19	0.78 ± 0.16
PHASE	257 ± 4	281 ± 7	86 ± 9	63 ± 18
***Controls PC-Δγ2***	**n = 6**	**n = 8**	**n = 6**	**n = 8**
FF (Hz)	54.75 ± 3.34	72.15 ± 5.53	0.96 ± 0.08	0.88 ± 0.07
AMPLITUDE (Hz)	18.86 ± 1.41	28.58 ± 3.73	0.94 ± 0.04	0.57 ± 0.04
PHASE	236 ± 14	273 ± 13	78 ± 14	68 ± 15
***Controls PC-ΔPP2B***	**n = 7**		**n = 7**	
FF (Hz)	65.66 ± 5.29		1.05 ± 0.10	
AMPLITUDE (Hz)	20.34 ± 3.03		1.42 ± 0.28	
PHASE	247 ± 15		60 ± 9	
***All Controls***	**n = 37**	**n = 27**	**n = 37**	**n = 27**
FF (Hz)	60.54 ± 2.07	72.82 ± 2.52	0.99 ± 0.04	0.88 ± 0.05
AMPLITUDE (Hz)	23.08 ± 2.02	37.95 ± 3.54	1.20 ± 0.08	0.62 ± 0.05
PHASE	247 ± 7	279 ± 6	85 ± 8	71 ± 9
**MODEL**	**runs = 30**	**runs = 30**		
FF (Hz)	56.93 ± 0.29	55.31 ± 0.4		
AMPLITUDE (Hz)	20.69 ± 0.35	27.37 ± 0.4		
PHASE	163 ± 1	160 ± 1		

All data are presented as mean ± SEM. Group sizes are denoted by *n* animals. “*Runs*” indicate the number of iterations of the model.

**Table 2:** Eye movement values during electrophysiological recordings before and after phase reversal adaptation in all control mice.

	Eye before	Eye after
***Control C57Bl/6***	***n***** = 14**	***n***** = 12**
GAIN	0.61 ± 0.02	0.36 ± 0.08
PHASE	43 ± 6	139 ± 8
***Control Δγ2***	***n***** = 6**	***n***** = 8**
GAIN	0.69 ± 0.04	0.39 ± 0.06
PHASE	38 ± 4	141 ± 7
***Control ΔKCC2***	**n = 10**	**n = 7**
GAIN	0.77 ± 0.10	0.28 ± 0.07
PHASE	37 ± 7	125 ± 7
***Control ΔPP2B***	**n = 7**	
GAIN	0.68 ± 0.10	
PHASE	38 ± 5	

All data are presented as mean ± SEM. Group sizes are denoted by *n* animals. Phase of the eye movements was quantified with respect to the stimulus rotation (table rotation).

**Table 3:** Eye movement values during electrophysiological recordings before and after phase reversal adaptation in all mutant mice.

	Eye before	Eye after
***GC-ΔKCC2***	**n = 5**	**n = 9**
GAIN	0.77 ± 0.06	0.35 ± 0.04
PHASE	27 ± 3	53 ± 2
***PC-Δγ2***	**n = 8**	**n = 10**
GAIN	0.60 ± 0.13	0.38 ± 0.06
PHASE	28 ± 5	66 ± 5
***PC-ΔKCC2***	**n = 9**	**n = 12**
GAIN	0.68 ± 0.16	0.70 ± 0.09
PHASE	36 ± 7	37 ± 4
***PC-ΔPP2B***	**n = 9**	**n = 12**
GAIN	0.73 ± 0.13	0.60 ± 0.09
PHASE	27 ± 4	26 ± 5
***GC - ΔCACNA1A***	**n = 9**	
GAIN	0.55 ± 0.12	
PHASE	52 ± 6	

All data are presented as mean ± SEM. Group sizes are denoted by *n* animals. Phase of the eye movements was quantified with respect to the stimulus rotation (table rotation).

**Table 4:** Purkinje cell activity during electrophysiological recordings before and after phase reversal adaptation in 5 mutant mice.

	SS naïve	SS after	Model naïve	Model after	CS naïve	CS after
***GC-ΔKCC2***	**n = 5**	**n = 9**	**runs = 30**	**runs = 30**	**n = 5**	**n = 9**
FF (Hz)	53.6 ± 10.5	77.8 ± 7.6	82.2 ± 0.5	99.0 ± 0.6	1.1 ± 0.0	1.3 ± 0.2
AMP (Hz)	12.2 ± 3.2	19.4 ± 4.0	20.8 ± 0.7	31.2 ± 0.8	1.2 ± 0.4	1.3 ± 0.3
PHASE	265 ± 15	244 ± 6	171 ± 1	166 ± 1	76 ± 4	65 ± 7
***PC-Δγ2***	**n = 8**	**n = 10**	**runs = 30**	**runs = 30**	**n = 8**	**n = 10**
FF (Hz)	44.9 ± 3.6	58.2 ± 3.7	66.5 ± 0.2	73.9 ± 0.3	0.9 ± 0.1	0.7 ± 0.1
AMP (Hz)	5.8 ± 3.4	20.2 ± 4.5	4.05 ± 0.3	11.1 ± 0.5	1.4 ± 0.1	0.8 ± 0.2
PHASE	243 ± 2	244 ± 7	128 ± 2.4	146 ± 1.0	83 ± 23	87 ± 12
***PC-ΔKCC2***	**n = 9**	**n = 12**	**runs = 30**	**runs = 30**	**n = 9**	**n = 12**
FF (Hz)	60.8 ± 7.4	61.6 ± 6.3	66.5 ± 0.2	73.9 ± 0.3	1.0 ± 0.1	0.9 ± 0.1
AMP (Hz)	6.4 ± 2.7	14.5 ± 2.9	4.1 ± 0.3	11.1 ± 0.5	1.1 ± 0.2	1.1 ± 0.2
PHASE	225 ± 14	224 ± 19	128 ± 2.4	146 ± 1.0	56 ± 8	72 ± 3
***PC-ΔPP2B***	**n = 9**	**n = 12**	**runs = 30**	**runs = 30**	**n = 9**	**n = 12**
FF (Hz)	47.4 ± 4.2	33.9 ± 3.4	0 ± 0	0 ± 0	1.1 ± 0.1	0.9 ± 0.1
AMP (Hz)	8.6 ± 5.0	15.8 ± 4.2	9.3 ± 0	9.3 ± 0	1.1 ± 0.2	1.1 ± 0.2
PHASE	253 ± 16	184 ± 33	181 ± 0	181 ± 0	192 ± 33	75 ± 12
***PC-ΔCACNA1A***	**n = 9**		**runs = 30**	**runs = 30**	**n = 9**	
FF (Hz)	60.5 ± 5.2		60.1 ± 0	60.1 ± 0	1.0 ± 0.1	
AMP (Hz)	8.6 ± 1.6		0.9 ± 0	0.9 ± 0	1.3 ± 0.2	
PHASE	223 ± 25		181 ± 0	181 ± 0	81 ± 12	

All data are presented as mean ± SEM. Group sizes are denoted by *n* animals. “*Runs*” indicate the number of iterations or the model.

**Table 5:** Purkinje cell activity values during electrophysiological recordings during VOR in *IO-ΔRobo3*.

	VOR		
	SS naïve	Model naïve	CS naïve
***Controls IO-ΔRobo3***	**n = 6**	**runs = 30**	**n = 6**
FF (Hz)	53,4 ± 5,7	56,9 ± 0,3	0,9 ± 0,1
AMPLITUDE (Hz)	30,3 ± 10,6	20,7 ± 0,3	1,8 ± 0,2
PHASE	163 ± 28	163 ± 1	77 ± 9
***IO-ΔRobo3***	**n = 30**	**runs = 30**	**n = 30**
FF (Hz)	58,8 ± 3,6	62,0 ± 2,0	1,0 ± 0,1
AMPLITUDE (Hz)	26,1 ± 3,9	3,0 ± 2,0	0,6 ± 0,1
PHASE	56 ± 5	19 ± 35	157 ± 13

All data are presented as mean ± SEM. Group sizes are denoted by *n* animals. “*Runs*” indicate the number of iterations or the model.
