# Supplementary material for: Modeled changes of cerebellar activity in mutant mice are predictive of their learning impairments
Source: Sci Rep. 2016 Nov 2;6:36131. doi: 10.1038/srep36131 (PMC5095348; doi:10.1038/srep36131)
Supplement: Supplementary Information [file srep36131-s1.pdf]

Supplementary Information for

Modeled changes of cerebellar activity in mutant mice are predictive of their learning impairments

Aleksandra Badura[1,2*], Claudia Clopath[3*], Martijn Schonewille[4] and Chris I. De Zeeuw[1,4]

Author for correspondence: a.badura@nin.knaw.nl

* These authors contributed equally to this work.

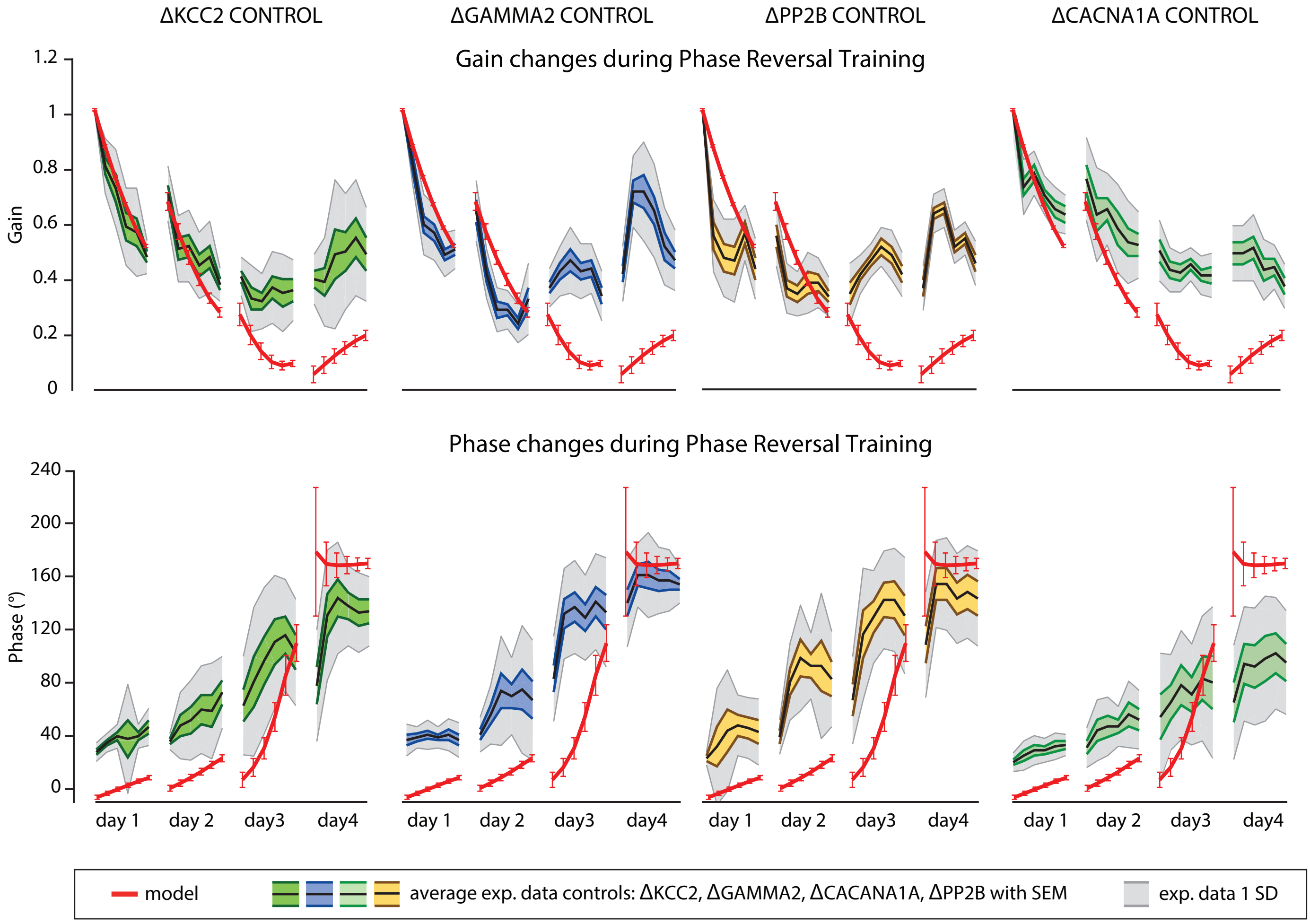

Supplementary Figure 1 | Experimental and modeled eye movements in control mice
Experimental and modeled eye movements in control mice displayed per genotype as a function of training time of VOR phase-reversal training. VOR values are measured in the dark; i.e. training is done in the light and eye measurement is done in the dark. Gain values (top panels) are normalized to the initial gain. Phase changes are depicted in bottom panels. Experimental data represent averages with SEM (color area) and SD (light grey area) of all control mice used in this study segregated by the genotype. Modeled changes are displayed for both gain and phase values with SD (red line).

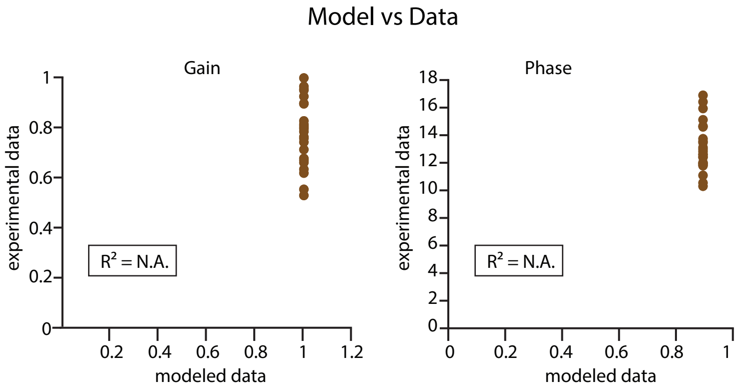

Supplementary Figure 2 | Experimental and modeled eye movements in *PC-ΔPP2B* mice
Linear regression plots displaying correlation between modeled and experimental data for gain (left) and phase (right) values.

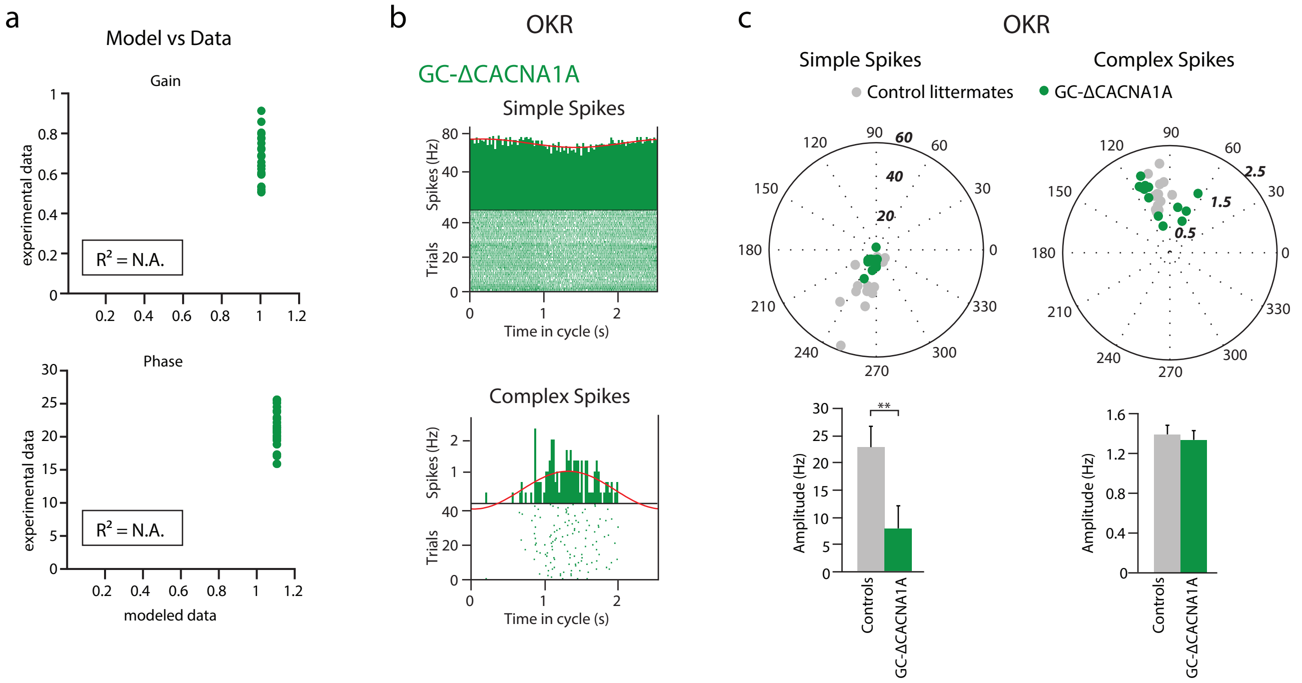

Supplementary Figure 3| Experimental and modeled eye movements in in *GC-ΔCACANA1A* mice
(**a**) Linear regression plots displaying correlation between modeled and experimental data for gain (top) and phase (bottom) values during the VOR phase reversal training. (**b**) Representative PSTH of floccular VA PC cell depicts SS and CS modulation (top and bottom panels, respectively) in response to visual stimulation (OKR) in *GC-ΔCACNA1A* mice. (**c**) Polar plots of SS and CS responses of *GC-ΔCACNA1A* and littermate control PCs (green and grey, respectively) during OKR stimulation. Each dot represents one cell. Corresponding histograms show that the modulation of SS but not CS during the OKR stimulation is attenuated in *GC-Δ CACNA1A* mice.

Supplementary Table 1. Gain values of the eye movements before and after phase reversal adaptation in all control mice.

GAIN		*Controls ΔKCC2 (PC + GC) n = 8*	*Controls PC-Δγ2 n = 10*	*Controls PC-ΔPP2B n = 8*	*Controls GC-ΔCACNA1A n = 8*	*All controls (average) n = 34*	*Model runs = 30*
**Day 1**	**t0**	1.00	1.00	1.00	1.00	1.00	1.00
	**t1**	0.81 ± 0.03	0.81 ± 0.03	0.56 ± 0.05	0.74 ± 0.03	0.72 ± 0.03	0.89 ± 0.00
	**t2**	0.73 ± 0.05	0.60 ± 0.03	0.48 ± 0.05	0.79 ± 0.03	0.64 ± 0.03	0.77 ± 0.00
	**t3**	0.59 ± 0.05	0.57 ± 0.03	0.47 ± 0.05	0.71 ± 0.02	0.59 ± 0.03	0.67 ± 0.00
	**t4**	0.57 ± 0.05	0.49 ± 0.02	0.57 ± 0.04	0.66 ± 0.03	0.54 ± 0.03	0.59 ± 0.00
	**t5**	0.48 ± 0.02	0.51 ± 0.02	0.44 ± 0.04	0.64 ± 0.03	0.53 ± 0.02	0.52 ± 0.00
**Day 2**	**t0**	0.71 ± 0.03	0.65 ± 0.04	0.56 ± 0.04	0.77 ± 0.05	0.69 ± 0.02	0.68 ± 0.01
	**t1**	0.51 ± 0.04	0.42 ± 0.03	0.37 ± 0.03	0.64 ± 0.06	0.46 ± 0.03	0.57 ± 0.01
	**t2**	0.52 ± 0.04	0.29 ± 0.03	0.35 ± 0.03	0.66 ± 0.04	0.47 ± 0.03	0.48 ± 0.00
	**t3**	0.45 ± 0.04	0.29 ± 0.02	0.39 ± 0.04	0.59 ± 0.06	0.46 ± 0.02	0.40 ± 0.00
	**t4**	0.48 ± 0.04	0.24 ± 0.02	0.39 ± 0.03	0.54 ± 0.05	0.43 ± 0.02	0.33 ± 0.00
	**t5**	0.38 ± 0.02	0.33 ± 0.04	0.34 ± 0.02	0.53 ± 0.04	0.40 ± 0.02	0.28 ± 0.00
**Day 3**	**t0**	0.41 ± 0.02	0.37 ± 0.02	0.35 ± 0.04	0.51 ± 0.04	0.41 ± 0.02	0.27 ± 0.01
	**t1**	0.33 ± 0.04	0.43 ± 0.03	0.42 ± 0.02	0.44 ± 0.03	0.40 ± 0.02	0.20 ± 0.01
	**t2**	0.32 ± 0.03	0.47 ± 0.04	0.47 ± 0.02	0.43 ± 0.03	0.44 ± 0.03	0.14 ± 0.01
	**t3**	0.37 ± 0.04	0.43 ± 0.03	0.52 ± 0.03	0.46 ± 0.02	0.47 ± 0.02	0.10 ± 0.00
	**t4**	0.35 ± 0.05	0.44 ± 0.03	0.49 ± 0.03	0.42 ± 0.02	0.41 ± 0.02	0.09 ± 0.00
	**t5**	0.36 ± 0.04	0.34 ± 0.03	0.42 ± 0.03	0.42 ± 0.03	0.39 ± 0.02	0.10 ± 0.00
**Day 4**	**t0**	0.40 ± 0.03	0.42 ± 0.03	0.37 ± 0.03	0.50 ± 0.04	0.43 ± 0.02	0.06 ± 0.01
	**t1**	0.39 ± 0.05	0.72 ± 0.04	0.64 ± 0.02	0.50 ± 0.04	0.47 ± 0.02	0.09 ± 0.01
	**t2**	0.49 ± 0.09	0.72 ± 0.06	0.66 ± 0.02	0.52 ± 0.04	0.49 ± 0.03	0.12 ± 0.00
	**t3**	0.50 ± 0.07	0.65 ± 0.05	0.52 ± 0.02	0.44 ± 0.04	0.47 ± 0.02	0.15 ± 0.00
	**t4**	0.55 ± 0.07	0.52 ± 0.05	0.55 ± 0.02	0.45 ± 0.03	0.51 ± 0.02	0.18 ± 0.00
	**t5**	0.49 ± 0.06	0.47 ± 0.03	0.46 ± 0.03	0.38 ± 0.03	0.44 ± 0.02	0.20 ± 0.00

All data are presented as mean ± SEM. Group sizes are denoted by *n* animals. "*Runs*" indicate the number of iterations of the model.

Supplementary Table 2. Gain values of the eye movements before and after phase reversal adaptation in all control mice.

PHASE		***Controls ΔKCC2 (PC + GC) n = 8***	***Controls PC-Δγ2 n = 10***	***Controls PC-ΔPP2B n = 8***	***Controls GC-ΔCACNA1A n = 8***	***All controls (average) n = 34***	***Model runs = 30***
**Day 1**	**t0**	28 ± 2	37 ± 4	22 ± 2	20 ± 2	29 ± 4	-7.0 ± 0.3
	**t1**	35 ± 2	39 ± 3	31 ± 15	25 ± 4	37 ± 5	-4.0 ± 0.3
	**t2**	40 ± 3	41 ± 3	43 ± 16	29 ± 4	42 ± 6	-1.0 ± 0.3
	**t3**	38 ± 14	39 ± 2	47 ± 8	29 ± 3	43 ± 6	2.1 ± 0.3
	**t4**	40 ± 3	41 ± 4	45 ± 8	32 ± 4	45 ± 7	5.1 ± 0.3
	**t5**	47 ± 5	37 ± 4	42 ± 9	33 ± 3	47 ± 6	7.8 ± 0.3
**Day 2**	**t0**	36 ± 2	41 ± 4	38 ± 5	31 ± 5	41 ± 5	-0.2 ± 0.4
	**t1**	48 ± 8	56 ± 7	80 ± 10	44 ± 8	67 ± 8	3.6 ± 0.4
	**t2**	52 ± 10	74 ± 13	98 ± 14	47 ± 8	73 ± 8	7.8 ± 0.4
	**t3**	60 ± 11	70 ± 9	92 ± 9	47 ± 5	77 ± 7	12.3 ± 0.5
	**t4**	59 ± 12	75 ± 15	92 ± 19	56 ± 9	83 ± 9	17.5 ± 0.5
	**t5**	73 ± 9	67 ± 14	82 ± 13	52 ± 8	79 ± 7	22.5 ± 0.5
**Day 3**	**t0**	63 ± 12	83 ± 10	66 ± 12	54 ± 17	70 ± 8	6.2 ± 1.0
	**t1**	81 ± 19	132 ± 11	116 ± 18	65 ± 13	114 ± 10	15.5 ± 1.2
	**t2**	97 ± 18	137 ± 11	129 ± 12	78 ± 15	121 ± 9	30.0 ± 1.5
	**t3**	111 ± 17	129 ± 10	142 ± 13	71 ± 13	128 ± 9	53.7 ± 2.0
	**t4**	116 ± 14	141 ± 11	142 ± 14	83 ± 16	125 ± 8	85.3 ± 2.8
	**t5**	103 ± 13	133 ± 13	130 ± 15	80 ± 20	116 ± 8	109.7 ± 2.5
**Day 4**	**t0**	78 ± 14	140 ± 10	108 ± 14	65 ± 15	79 ± 8	178.9 ± 8.9
	**t1**	131 ± 16	161 ± 8	154 ± 12	94 ± 15	131 ± 8	169.6 ± 3.0
	**t2**	144 ± 14	161 ± 10	154 ± 12	92 ± 16	131 ± 8	168.6 ± 1.7
	**t3**	138 ± 10	157 ± 8	143 ± 12	98 ± 17	127 ± 7	168.8 ± 1.2
	**t4**	133 ± 10	157 ± 7	148 ± 13	102 ± 15	129 ± 7	169.3 ± 0.9
	**t5**	134 ± 9	154 ± 4	143 ± 13	95 ± 14	124 ± 7	170.0 ± 0.7

All data are presented as mean ± SEM. Group sizes are denoted by *n* animals. "*Runs*" indicate the number of iterations of the model.

Supplementary Table 3. R^2 values of the linear regression for the eye movements during phase reversal training between model and control mice

GAIN	*Controls ΔKCC2 (PC+GC) n = 8*	*Controls PC-Δγ2 n = 10*	*Controls PC-ΔPP2B n = 8*	*Controls GC-ΔCACNA1A n = 8*	*All controls (average) n = 34*
**Day 1**	0.98	0.91	0.59	0.81	0.87
**Day 2**	0.80	0.69	0.51	0.91	0.72
**Day 3**	0.23	0.04	0.70	0.64	0.05
**Day 4**	0.70	0.01	0.01	0.68	0.08
**Total training**	0.78	0.22	0.14	0.89	0.68

PHASE	*Controls ΔKCC2 (PC+GC) n = 8*	*Controls PC-Δγ2 n = 10*	*Controls PC-ΔPP2B n = 8*	*Controls GC-ΔCACNA1A n = 8*	*All controls (average) n = 34*
**Day 1**	0.86	0.03	0.66	0.93	0.86
**Day 2**	0.92	0.52	0.33	0.73	0.67
**Day 3**	0.59	0.31	0.42	0.64	0.29
**Day 4**	0.99	0.92	0.92	0.90	0.98
**Total training**	0.76	0.74	0.62	0.74	0.55

All data are presented as mean ± SEM. Group sizes are denoted by *n* animals. “Total” depicts R^2 values for the entire training.

Supplementary Table 4. Gain values of the eye movements before and after phase reversal adaptation in all mutant mice.

GAIN		*GC-ΔKCC2*		*PC-Δγ2*		*PC-ΔKCC2*	
		Experimental (n = 8)	***Model (runs = 30)***	***Experimental (n = 9)***	***Model (runs = 30)***	***Experimental (n = 8)***	***Model (runs = 30)***
**Day 1**	**t0**	1.00	1.00	1.00	1.00	1.00	1.00
	**t1**	0.71 ± 0.03	0.91 ± 0.00	0.92 ± 0.03	0.90 ± 0.00	0.98 ± 0.04	0.90 ± 0.00
	**t2**	0.66 ± 0.05	0.82 ± 0.00	0.81 ± 0.03	0.80 ± 0.00	0.88 ± 0.05	0.80 ± 0.00
	**t3**	0.75 ± 0.03	0.75 ± 0.00	0.75 ± 0.04	0.72 ± 0.00	0.78 ± 0.03	0.72 ± 0.00
	**t4**	0.64 ± 0.03	0.68 ± 0.00	0.69 ± 0.04	0.65 ± 0.00	0.82 ± 0.04	0.65 ± 0.00
	**t5**	0.65 ± 0.03	0.63 ± 0.00	0.71 ± 0.02	0.60 ± 0.00	0.67 ± 0.04	0.60 ± 0.00
**Day 2**	**t0**	0.82 ± 0.04	0.88 ± 0.01	0.96 ± 0.04	0.83 ± 0.01	0.85 ± 0.04	0.83 ± 0.01
	**t1**	0.67 ± 0.04	0.78 ± 0.01	0.81 ± 0.02	0.71 ± 0.01	0.81 ± 0.05	0.71 ± 0.01
	**t2**	0.67 ± 0.04	0.68 ± 0.01	0.76 ± 0.04	0.61 ± 0.01	0.69 ± 0.05	0.61 ± 0.01
	**t3**	0.63 ± 0.05	0.61 ± 0.01	0.67 ± 0.02	0.54 ± 0.00	0.66 ± 0.04	0.54 ± 0.00
	**t4**	0.65 ± 0.04	0.56 ± 0.01	0.62 ± 0.03	0.49 ± 0.00	0.67 ± 0.05	0.49 ± 0.00
	**t5**	0.62 ± 0.04	0.52 ± 0.01	0.59 ± 0.01	0.46 ± 0.00	0.62 ± 0.05	0.46 ± 0.00
**Day 3**	**t0**	0.71 ± 0.04	0.74 ± 0.01	0.82 ± 0.02	0.65 ± 0.01	0.85 ± 0.05	0.65 ± 0.01
	**t1**	0.55 ± 0.04	0.63 ± 0.01	0.64 ± 0.02	0.53 ± 0.01	0.76 ± 0.05	0.53 ± 0.01
	**t2**	0.58 ± 0.03	0.54 ± 0.01	0.63 ± 0.02	0.43 ± 0.01	0.65 ± 0.05	0.43 ± 0.01
	**t3**	0.55 ± 0.03	0.47 ± 0.01	0.52 ± 0.02	0.37 ± 0.00	0.61 ± 0.04	0.37 ± 0.00
	**t4**	0.57 ± 0.02	0.43 ± 0.01	0.47 ± 0.02	0.33 ± 0.00	0.60 ± 0.05	0.33 ± 0.00
	**t5**	0.44 ± 0.02	0.41 ± 0.01	0.43 ± 0.02	0.30 ± 0.00	0.53 ± 0.04	0.30 ± 0.00
**Day 4**	**t0**	0.57 ± 0.02	0.62 ± 0.02	0.56 ± 0.02	0.47 ± 0.01	0.62 ± 0.05	0.47 ± 0.01
	**t1**	0.44 ± 0.02	0.52 ± 0.01	0.38 ± 0.03	0.36 ± 0.01	0.53 ± 0.05	0.36 ± 0.01
	**t2**	0.42 ± 0.03	0.44 ± 0.01	0.41 ± 0.02	0.29 ± 0.01	0.52 ± 0.04	0.29 ± 0.01
	**t3**	0.50 ± 0.03	0.38 ± 0.01	0.38 ± 0.02	0.24 ± 0.00	0.48 ± 0.03	0.24 ± 0.00
	**t4**	0.52 ± 0.03	0.34 ± 0.01	0.32 ± 0.02	0.20 ± 0.00	0.47 ± 0.04	0.20 ± 0.00
	**t5**	0.44 ± 0.03	0.32 ± 0.01	0.35 ± 0.02	0.18 ± 0.00	0.43 ± 0.05	0.18 ± 0.00

Supplementary Table 4. (Continued)

GAIN		*PC-ΔPP2B*		*GC-ΔCACNA1A*	
		Experimental (n = 8)	***Model (runs = 30)***	***Experimental (n = 6)***	***Model (runs = 30)***
**Day 1**	**t0**	1.00	1.00	1.00	1.00
	**t1**	0.93 ± 0.08	1.00 ± N/A	0.94 ± 0.04	1.00 ± N/A
	**t2**	0.95 ± 0.04	1.00 ± N/A	0.87 ± 0.06	1.00 ± N/A
	**t3**	0.80 ± 0.07	1.00 ± N/A	0.82 ± 0.07	1.00 ± N/A
	**t4**	0.68 ± 0.08	1.00 ± N/A	0.88 ± 0.05	1.00 ± N/A
	**t5**	0.71 ± 0.04	1.00 ± N/A	0.72 ± 0.06	1.00 ± N/A
**Day 2**	**t0**	0.90 ± 0.04	1.00 ± N/A	0.85 ± 0.04	1.00 ± N/A
	**t1**	0.97 ± 0.06	1.00 ± N/A	0.79 ± 0.04	1.00 ± N/A
	**t2**	0.95 ± 0.04	1.00 ± N/A	0.75 ± 0.03	1.00 ± N/A
	**t3**	0.90 ± 0.05	1.00 ± N/A	0.75 ± 0.05	1.00 ± N/A
	**t4**	0.83 ± 0.07	1.00 ± N/A	0.68 ± 0.05	1.00 ± N/A
	**t5**	0.81 ± 0.04	1.00 ± N/A	0.65 ± 0.04	1.00 ± N/A
**Day 3**	**t0**	0.74 ± 0.05	1.00 ± N/A	0.85 ± 0.06	1.00 ± N/A
	**t1**	0.76 ± 0.07	1.00 ± N/A	0.70 ± 0.04	1.00 ± N/A
	**t2**	0.76 ± 0.07	1.00 ± N/A	0.65 ± 0.04	1.00 ± N/A
	**t3**	0.80 ± 0.07	1.00 ± N/A	0.70 ± 0.05	1.00 ± N/A
	**t4**	0.81 ± 0.02	1.00 ± N/A	0.58 ± 0.04	1.00 ± N/A
	**t5**	0.78 ± 0.05	1.00 ± N/A	0.66 ± 0.05	1.00 ± N/A
**Day 4**	**t0**	0.53 ± 0.08	1.00 ± N/A	0.76 ± 0.08	1.00 ± N/A
	**t1**	0.55 ± 0.09	1.00 ± N/A	0.79 ± 0.09	1.00 ± N/A
	**t2**	0.67 ± 0.06	1.00 ± N/A	0.58 ± 0.05	1.00 ± N/A
	**t3**	0.66 ± 0.06	1.00 ± N/A	0.56 ± 0.06	1.00 ± N/A
	**t4**	0.63 ± 0.06	1.00 ± N/A	0.55 ± 0.08	1.00 ± N/A
	**t5**	0.62 ± 0.06	1.00 ± N/A	0.57 ± 0.05	1.00 ± N/A

All data are presented as mean ± SEM. Group sizes are denoted by *n* animals. "*Runs*" indicate the number of iterations of the model.

Supplementary Table 5. Phase values of the eye movements before and after phase reversal adaptation in all mutant mice.

**PHASE**		**GC-ΔKCC2**		**PC-Δγ2**		**PC-ΔKCC2**	
		**Experimental (n = 8)**	**Model (runs = 30)**	**Experimental (n = 9)**	**Model (runs = 30)**	**Experimental (n = 8)**	**Model (runs = 30)**
**Day 1**	**t0**	25 ± 1	-4.4 ± 0.4	19 ± 2	-5.1 ± 0.3	23 ± 1	-5.1 ± 0.3
	**t1**	35 ± 2	-1.4 ± 0.3	17 ± 2	-2.1 ± 0.3	25 ± 2	-2.1 ± 0.3
	**t2**	38 ± 3	0.8 ± 0.3	17 ± 2	0.6 ± 0.3	28 ± 2	0.6 ± 0.3
	**t3**	37 ± 3	2.8 ± 0.3	20 ± 1	3.1 ± 0.3	28 ± 1	3.1 ± 0.3
	**t4**	42 ± 3	4.8 ± 0.3	22 ± 2	5.2 ± 0.3	28 ± 1	5.2 ± 0.3
	**t5**	40 ± 3	6.2 ± 0.3	22 ± 2	6.9 ± 0.2	27 ± 1	6.9 ± 0.2
**Day 2**	**t0**	28 ± 2	-3.1 ± 0.5	18 ± 1	-1.8 ± 0.4	25 ± 1	-1.8 ± 0.4
	**t1**	37 ± 4	0.4 ± 0.5	19 ± 1	1.9 ± 0.4	26 ± 2	1.9 ± 0.4
	**t2**	39 ± 3	3.3 ± 0.4	20 ± 2	5.2 ± 0.3	29 ± 2	5.2 ± 0.3
	**t3**	39 ± 4	6.0 ± 0.4	20 ± 2	8.1 ± 0.3	32 ± 3	8.1 ± 0.3
	**t4**	44 ± 5	8.2 ± 0.4	20 ± 2	10.3 ± 0.3	28 ± 3	10.3 ± 0.3
	**t5**	44 ± 5	9.6 ± 0.4	23 ± 1	11.9 ± 0.3	29 ± 2	11.9 ± 0.3
**Day 3**	**t0**	29 ± 2	-1.7 ± 0.4	19 ± 1	-1.0 ± 0.4	25 ± 2	-1.0 ± 0.4
	**t1**	42 ± 4	2.9 ± 0.4	23 ± 1	4.3 ± 0.4	31 ± 2	4.3 ± 0.4
	**t2**	46 ± 5	6.9 ± 0.3	26 ± 2	9.4 ± 0.5	36 ± 3	9.4 ± 0.5
	**t3**	52 ± 9	10.4 ± 0.4	26 ± 2	13.7 ± 0.4	33 ± 3	13.7 ± 0.4
	**t4**	54 ± 10	13.1 ± 0.4	27 ± 2	17.5 ± 0.4	34 ± 4	17.5 ± 0.4
	**t5**	62 ± 13	15.1 ± 0.5	30 ± 2	20.2 ± 0.4	37 ± 5	20.2 ± 0.4
**Day 4**	**t0**	32 ± 5	-1.0 ± 0.6	25 ± 2	-1.5 ± 0.6	28 ± 2	-1.5 ± 0.6
	**t1**	55 ± 8	3.7 ± 0.5	30 ± 5	5.0 ± 0.7	40 ± 6	5.0 ± 0.7
	**t2**	59 ± 8	8.2 ± 0.6	30 ± 3	12.1 ± 0.8	35 ± 3	12.1 ± 0.8
	**t3**	56 ± 6	12.4 ± 0.6	33 ± 6	19.4 ± 0.9	35 ± 3	19.4 ± 0.9
	**t4**	56 ± 6	15.9 ± 0.6	37 ± 4	25.8 ± 1.0	38 ± 5	25.8 ± 1.0
	**t5**	61 ± 7	18.3 ± 0.7	40 ± 8	30.9 ± 1.0	39 ± 4	30.9 ± 1.0

Supplementary Table 5. (Continued)

PHASE		PC-ΔPP2B Experimental (n = 8)	PC-ΔPP2B Model (runs = 30)	GC-ΔCACNA1A Experimental (n = 6)	GC-ΔCACNA1A Model (runs = 30)
**Day 1**	**t0**	13 ± 2	0.9 ± N/A	16 ± 1	1.1 ± N/A
	**t1**	13 ± 1	0.9 ± N/A	18 ± 1	1.1 ± N/A
	**t2**	15 ± 1	0.9 ± N/A	19 ± 1	1.1 ± N/A
	**t3**	15 ± 2	0.9 ± N/A	21 ± 1	1.1 ± N/A
	**t4**	14 ± 3	0.9 ± N/A	22 ± 1	1.1 ± N/A
	**t5**	15 ± 2	0.9 ± N/A	23 ± 1	1.1 ± N/A
**Day 2**	**t0**	10 ± 1	0.9 ± N/A	16 ± 1	1.1 ± N/A
	**t1**	12 ± 2	0.9 ± N/A	20 ± 1	1.1 ± N/A
	**t2**	14 ± 1	0.9 ± N/A	20 ± 1	1.1 ± N/A
	**t3**	15 ± 1	0.9 ± N/A	21 ± 2	1.1 ± N/A
	**t4**	15 ± 1	0.9 ± N/A	23 ± 1	1.1 ± N/A
	**t5**	15 ± 1	0.9 ± N/A	24 ± 1	1.1 ± N/A
**Day 3**	**t0**	11 ± 2	0.9 ± N/A	17 ± 1	1.1 ± N/A
	**t1**	13 ± 2	0.9 ± N/A	20 ± 1	1.1 ± N/A
	**t2**	15 ± 1	0.9 ± N/A	20 ± 1	1.1 ± N/A
	**t3**	15 ± 2	0.9 ± N/A	21 ± 2	1.1 ± N/A
	**t4**	17 ± 1	0.9 ± N/A	24 ± 1	1.1 ± N/A
	**t5**	17 ± 1	0.9 ± N/A	25 ± 2	1.1 ± N/A
**Day 4**	**t0**	9 ± 3	0.9 ± N/A	21 ± 1	1.1 ± N/A
	**t1**	11 ± 4	0.9 ± N/A	22 ± 2	1.1 ± N/A
	**t2**	13 ± 1	0.9 ± N/A	25 ± 3	1.1 ± N/A
	**t3**	15 ± 1	0.9 ± N/A	25 ± 2	1.1 ± N/A
	**t4**	15 ± 2	0.9 ± N/A	23 ± 5	1.1 ± N/A
	**t5**	16 ± 1	0.9 ± N/A	21 ± 4	1.1 ± N/A

All data are presented as mean ± SEM. Group sizes are denoted by *n* animals. "*Runs*" indicate the number of iterations of the model.

Supplementary Table 6. R^2 values of the linear regression for the eye movements during phase reversal training between model and control mice

GAIN	*GC-ΔKCC2* n = 8	*PC-Δγ2* n = 9	*PC-ΔKCC2* n = 8	*PC-ΔPP2B* n = 8	*GC-ΔCACNA1A* n = 6
**Day 1**	0.60	0.96	0.88	-	-
**Day 2**	0.78	0.99	0.93	-	-
**Day 3**	0.66	0.96	0.97	-	-
**Day 4**	0.15	0.78	0.94	-	-
**Total training**	0.77	0.95	0.94	-	-

PHASE	*GC-ΔKCC2* n = 8	*PC-Δγ2* n = 9	*PC-ΔKCC2* n = 8	*PC-ΔPP2B* n = 8	*GC-ΔCACNA1A* n = 6
**Day 1**	0.81	0.46	0.68	-	-
**Day 2**	0.89	0.74	0.52	-	-
**Day 3**	0.95	0.93	0.71	-	-
**Day 4**	0.60	0.93	0.38	-	-
**Total training**	0.81	0.75	0.64	-	-

All data are presented as mean ± SEM. Group sizes are denoted by *n* animals.

Supplementary Table 7. Purkinje cell SS regularity.

	Phase Reversal Adaptation	
	**SS naïve**	**SS after**
Controls Black6	**n = 14**	**n = 12**
CV	0,62 ± 0,04	0,87 ± 0,11
CV2	0,43 ± 0,02	0,50 ± 0,03
Controls PC-Δγ2	**n = 6**	**n = 8**
CV	0,52 ± 0,03	0,69 ± 0,03
CV2	0,48 ± 0,04	0,51 ± 0,03
Controls ΔKCC2	**n = 10**	**n = 7**
CV	0,55 ± 0,05	0,73 ± 0,13
CV2	0,44 ± 0,03	0,55 ± 0,06
PC-Δγ2	**n = 8**	**n = 10**
CV	0,40 ± 0,02	0,54 ± 0,04
CV2	0,32 ± 0,02	0,36 ± 0,02
PC-ΔKCC2	**n = 9**	**n = 12**
CV	0,58 ± 0,06	0,68 ± 0,17
CV2	0,38 ± 0,02	0,34 ± 0,02

All data are presented as mean ± SEM. Group sizes are denoted by *n* animals. "*Runs*" indicate the number of iterations of the model.
